# The tokophobia severity scale: a psychometric multicountry study with childbearing-age women

**DOI:** 10.1007/s00737-026-01736-9

**Published:** 2026-06-18

**Authors:** Daniela Fidalgo, Matilde Sousa, Cláudia Sousa, Julie Jomeen, Paulina Pawlicka, Olga Riklikienė, Diogo Lamela, Kathleen Baird, Barbara Baranowska, Gabija Jarašiūnaitė-Fedosejeva, Paulina Mistelska, Inês Jongenelen, Tiago Miguel Pinto, Raquel Costa

**Affiliations:** 1 HEI‐Lab: Digital Human‐Environment Interaction Labs, Universidade Lusófona, Porto, Portugal; 2 Insight - Piaget Research Center for Ecological Human Development, Porto, Portugal; 3 Faculty of Health, Southern Cross University, Queensland, Australia; 4 Department of Social Sciences, Institute of Psychology, University of Gdańsk, Gdańsk, Poland; 5 Nursing Department, Faculty of Nursing, Lithuanian University of Health Sciences, Kaunas, Lithuania; 6 School of Nursing and Midwifery, Faculty of Health, University of Technology Sydney, Sydney, Australia; 7 Department of Midwifery, Centrum Medyczne Kształcenia Podyplomowego, Warsaw, Poland; 8 Department of Psychology, Faculty of Social Sciences, Vytautas Magnus University, Kaunas, Lithuania

**Keywords:** Tokophobia, Childbirth, Pregnancy,depression, Anxiety

## Abstract

**Purpose:**

Tokophobia refers to a clinically significant fear of childbirth that affects women across the reproductive lifespan. Given its prevalence and association with adverse mental health, obstetric, and reproductive decision-making outcomes, valid and reliable measures are essential to identify women at risk of tokophobia.

**Methods:**

Tokophobia Severity Scale (TSS) psychometric properties were analyzed in nulliparous non-pregnant, pregnant, and women in the postpartum using data from cross-sectional and longitudinal studies conducted in four countries: Australia, Lithuania, Poland, and Portugal. Sample (*n* = 1,585) comprised nulliparous non-pregnant (Poland, *n* = 559), pregnant (Lithuania, *n* = 197, Portugal, *n* = 353), and women at two months postpartum (Australia, *n* = 292; Lithuania, *n* = 184). Participants completed the TSS, and self-reported questionnaires on sociodemographic, obstetric, fetal/neonatal, mental health-related characteristics, depressive and anxiety symptoms, birth trauma perception, and another measure of fear of childbirth (FOC).

**Results:**

Results supported a three-factor model: fear, coping, and intrusive thoughts. Invariance was found across childbearing-age periods and countries. Cronbach’s alpha and McDonald’s omega coefficients for the TSS total score were 0.89-0.90. Known-group validity was established with differences in sociodemographic, obstetric, fetal/neonatal, and mental health-related characteristics. Concurrent validity was found with depressive and anxiety symptoms, and birth trauma perception. Convergent validity was found with another FOC measure. TSS scores in pregnancy predicted elective cesarean section, birth trauma perception, and postpartum depressive and anxiety symptoms. TSS discriminates women with or without clinically significant tokophobia symptoms in childbearing-age periods and countries.

**Conclusions:**

TSS is a psychometrically strong measure that can be used to screen clinically significant tokophobia symptoms.

**Supplementary Information:**

The online version contains supplementary material available at https://doi.org/10.1007/s00737-026-01736-9.

## Introduction

Tokophobia is an under-recognized and under-prioritized mental health issue (Wootton et al. 2020). It is defined as a pathological fear of childbirth that affects childbearing-age women (Hofberg and Brockington 2000; Wijma et al. 1998). The estimated prevalence of tokophobia (i.e., clinically significant fear of childbirth) is 16% in nulliparous women, ranges from 7 to 25% in primiparous women, and from 7 to 16% in multiparous women (Kanellopoulos and Gourounti 2022; O’Connell et al. 2017). Moreover, Fairbrother et al. (2022) found that 11% of pregnant women report severe fear of childbirth, and 2% experienced a *phobic-like* fear, characterized by features similar to a specific phobia without necessarily meeting full diagnostic criteria. It falls under the diagnostic criteria of unspecified Phobic Anxiety Disorder by the International Classification of Diseases-11 (ICD-11; World Health Organization [WHO], 2019), and of Specific Phobia by the Diagnostic and Statistical Manual of Mental Disorders (DSM-5-TR; American Psychiatric Association [APA], 2022), including symptoms of fear, anxiety, or avoidance of objects or situations, to a degree that is out of proportion to the actual danger (APA, 2022). However, some individuals may experience significant fear of childbirth without meeting the full diagnostic criteria for Specific Phobia or Unspecified Phobic Anxiety Disorder. Anxiety in general is often marked by pervasive and future-oriented worry across multiple life domains (APA, 2022), whereas tokophobia is distinguished by worry focusing specifically on childbirth (WHO, 2019). Tokophobia can be classified as primary or secondary. Primary tokophobia refers to a pathological fear of childbirth without prior experience of childbirth or trauma related to childbirth (Hofberg and Brockington 2000), whereas secondary tokophobia takes place after exposure to potentially traumatic obstetric events, such as miscarriage, termination of pregnancy, prolonged labor, intense pain, or stillbirth (Denker et al. 2019; Hofberg and Brockington 2000; Hofberg and Ward 2004).

The etiology of tokophobia has been linked to several sociodemographic, obstetric, and mental health-related factors such as older age (Räisänen et al. 2014), higher education (Dal Moro et al. 2023), socio-economic disadvantage (Kim et al. 2018), nulliparity (Rouhe et al. 2009), previous miscarriage (Smorti et al. 2021), obstetric and fetal/neonatal complications (Vaajala et al. 2023), and with previous or current mental health problems, such as anxiety or depressive symptoms (Dencker et al. 2019; Grundström et al. 2022; Storksen et al. 2012). Additionally, in nulliparous women higher birth-related knowledge was associated with lower tokophobia symptoms (Antić et al. 2019).

Tokophobia symptoms can negatively affect women’s reproductive choices, mental health (distress, anxiety, and depressive symptoms), overall quality of life, mother-infant bonding, and other interpersonal relationships (Kitamura et al. 2024; Molgora et al. 2020; Pascal et al. 2023; Rublein and Muschalla 2022; Ryding et al. 2015; Seefeld et al. 2022). Women with tokophobia are more likely to prefer elective cesarean section (vs. vaginal birth), pain relief (vs. no pain relief), or epidural analgesia in a vaginal birth, compared to those without tokophobia (Sitras et al. 2017; Hendrix et al. 2022).

Given the deleterious and potentially enduring consequences, the value of identifying tokophobia in childbearing women is evident. In response, several assessment measures were developed, including the Tokophobia Severity Scale (TSS). The TSS) items were developed by experts in the assessment of anxiety-related disorders and selected with the support of empirical data, using descriptive statistics, factor structure, and inter-item correlation (Wootton et al. 2020). The final 13-item self-report version of the scale assesses both the cognitive (e.g., worry; “I worry about medical complications during pregnancy and/or childbirth”) and behavioral (e.g., avoidance; “I avoid talking about pregnancy, children and childbirth because of my fears”) aspects of tokophobia. Compared to other measures of fear of childbirth (e.g., Wijma Delivery Expectancy Questionnaire [Wijma et al. 1998 ], Fear of Birth Scale [FOBS; Haines et al. 2011 ]), the TSS has the particularity of focusing on both the cognitive and behavioral aspects of tokophobia, particularly worry, which is aligned with the diagnostic criteria proposed by the ICD-11 and the DSM-5-TR. Its psychometric properties were tested in a sample of Australian nulliparous non-pregnant women (Wootton et al. 2020), showing good internal consistency (Cronbach’s alpha = 0.93) and convergent and divergent validity with other measures of fear of childbirth and depressive symptoms, respectively. The factor structure was also tested (Martin et al. 2021), and a three-factor model - Fear, Coping, and Intrusive thoughts - with 13 or 10 items (i.e. excluding items 1, 2, and 10 due to floor effects) offered a better fit than the unidimensional model (Wootton et al. 2020). As far as we are aware, evidence on the TSS’s psychometric properties comes only from studies with pregnant women in Iran (Zardkoohi et al. 2023), a country with cultural, linguistic, and reproductive healthcare contexts markedly different from those of high-income countries such as Portugal, Australia, Poland, and Lithuania. Other validations were conducted with nulliparous non-pregnant women from Australia or Lebanon (Gerges et al. 2024; Wootton et al. 2020). Evidence is lacking in most countries for women of childbearing age, and there are psychometric properties that have not yet been tested (e.g., criterion validity, predictive validity, or clinical validity).

Assessing the psychometric properties of the TSS in childbearing-age women using multi-country data is a step forward for effective screening of functional impairment due to primary or secondary tokophobia in health care services. Given the TSS focus on worry - a transdiagnostic features of many mental health disorders (Akbari et al. 2018) - it has also the potential to predict further mental health problems in the postpartum period. This study aimed to analyze the psychometric properties of the TSS in nulliparous non-pregnant women, pregnant women, and women in the postpartum period using data from four countries: Australia, Lithuania, Poland, and Portugal. Specific aims were to analyze TSS’ (1) factor structure and measurement invariance; (2) reliability; (3) criterion validity (known groups and concurrent); (4) convergent validity; (5) predictive validity; and (6) clinical validity.

## Methods

### Participants

The sample included 1,585 women, of whom 559 nulliparous non-pregnant women (35.3%; data from Poland), 550 pregnant women (34.7%; data from Lithuania *n* = 197 and Portugal *n* = 353), and 476 women in the postpartum period (30%; data from Australia *n* = 292 and Lithuania *n* = 184). Nulliparous non-pregnant women, pregnant women, and women in the postpartum period mean ages were 20.80, 32.07, and 31.70 years (*SD* = 2.78, range = 17–42; *SD* = 5.08, range 20–46; *SD* = 5.05, range = 18–47), respectively. Table 1 depicts sociodemographic characteristics and Supplementary Table 1 depicts detailed information on childbirth perception for non-pregnant women.

**Table 1:** Sociodemographic, obstetric, and fetal/neonatal characteristics, and mental health-related characteristics (*N* = 1585)

		Nulliparous non-pregnant women	Pregnant women			Postpartum women		
		Poland	Lithuania	Portugal	Total	Australia	Lithuania	Total
		*n* = 559 *(%)*	*n* = 197 *(%)*	*n* = 353 *(%)*	*n* = 550 *(%)*	*n* = 292 *(%)*	*n* = 184 *(%)*	*n* = 476 *(%)*
Sociodemographic characteristics								
Age	*M (SD)*	20.80 (2.78)	30.35 (4.52)	33.05 (5.12)	32.07 (5.08)	32.42 (5.26)	30.59 (4.50)	31.70 (5.05)
	*Missings*	70 (12.5)	0 (0.0)	7 (2.0)	7 (1.3)	7 (2.4)	0 (0.0)	7 (1.5)
Ethnicity	Ethnic majority	-	-	286 (87.2)	286 (87.2)	-	-	-
	Ethnic minority	-	-	9 (2.7)	9 (2.7)	-	-	-
	Not sure	-	-	33 (10.1)	33 (10.1)	-	-	-
	*Missings*	-	-	25 (7.1)	222 (40.4)	-	-	-
Perception of Income	Above average	95 (17.0)	93 (47.2)	68 (20.2)	161 (30.2)	51 (17.8)	61 (33.2)	112 (23.8)
	Average	437 (78.2)	101 (51.3)	231 (68.8)	332 (62.3)	195 (67.9)	120 (65.2)	315 (66.9)
	Below average	27 (4.8)	3 (1.5)	37 (11.0)	40 (7.5)	41 (14.3)	3 (1.6)	44 (9.3)
	*Missings*	0 (0.0)	0 (0.0)	17 (4.8)	17 (3.1)	5 (1.7)	0 (0.0)	5 (1.1)
Marital status	Married/cohabiting	215 (38.5)	189 (96.0)	326 (94.2)	515 (94.9)	260 (90.3)	184 (100.0)	444 (94.1)
	Other	344 (61.5)	8 (4.0)	20 (5.9)	28 (5.2)	28 (9.7)	0 (0.0)	28 (6.0)
	*Missings*	0 (0.0)	0 (0.0)	7 (2.0)	7 (1.3)	4 (1.4)	0 (0.0)	4 (0.8)
Education	Higher education	0 (0.0)	168 (85.3)	219 (62.8)	387 (70.9)	212 (73.9)	130 (70.7)	342 (72.6)
	Other	559 (100.0)	29 (14.7)	130 (37.2)	159 (29.1)	75 (26.1)	54 (29.4)	129 (27.3)
	*Missings*	0 (0.0)	0 (0.0)	4 (1.1)	4 (0.7)	5 (1.7)	0 (0.0)	5 (1.1)
Obstetric and fetal/neonatal characteristics								
Parity	Primiparous	-	133 (67.5)	210 (59.7)	343 (62.5)	135 (46.9)	109 (59.2)	244 (51.7)
	Multiparous	-	64 (32.5)	142 (40.3)	206 (37.5)	153 (53.1)	75 (40.8)	228 (48.3)
	*Missings*	-	0 (0.0)	1 (0.3)	1 (0.2))	4 (1.4)	0 (0.0)	4 (0.8)
Previous pregnancy loss	Yes	-	43 (21.8)	97 (27.7)	140 (25.6)	77 (50.3)	51 (27.9)	128 (38.1)
	No	-	154 (78.2)	253 (72.3)	407 (74.4)	76 (49.7)	132 (72.1)	208 (61.9)
	*Missings*	-	0 (0.0)	3 (0.8)	3 (0.5)	139 (47.6)	1 (0.5)	140 (29.4)
Obstetric complications	Major	-	4 (2.0)	20 (5.7)	24 (4.4)	27 (9.2)	15 (8.2)	42 (8.8)
	Minor	-	55 (27.9)	120 (34.4)	175 (32.1)	125 (42.8)	44 (23.9)	169 (35.5)
	None	-	138 (70.1)	209 (59.9)	347 (63.6)	140 (47.9)	125 (67.9)	265 (55.7)
	*Missings*	-	0 (0.0)	4 (1.1)	4 (0.7)	0 (0.0)	0 (0.0)	0 (0.0)
Fetal/ neonatal complications	Major	-	-	11 (3.1)	11 (3.1)	15 (5.2)	-	15 (5.2)
	Minor	-	-	29 (8.3)	29 (8.3)	70 (24.1)	-	70 (24.1)
	None	-	-	311 (88.6)	83.3311 (88.6)	206 (70.8)	-	206 (70.8)
	*Missings*	-	-	2 (0.6)	199 (36.2)	1 (0.3)	-	185 (38.9)
Type of birth	Vaginal	-	-	-	-	137 (46.9)	138 (75.0)	275 (57.8)
	Assisted vaginal	-	-	-	-	22 (7.5)	8 (4.3)	30 (6.3)
	Emergency Caesarean	-	-	-	-	50 (17.1)	28 (15.2)	78 (16.4)
	Elective Cesarean	-	-	-	-	83 (28.4)	10 (5.4)	93 (19.5)
	Missings	-	-	-	-	0 (0.0)	0 (0.0)	0 (0.0)
Mental health-related factors								
Potentially traumatic lifetime events	Yes	97 (52.7)	90 (45.7)	97 (29.2)	187 (35.3)	-	179 (61.3)	276 (58.0)
	No	87 (47.3)	107 (54.3)	235 (70.8)	342 (64.7)	-	113 (38.7)	200 (42.0)
	*Missings*	0 (0.0)	0 (0.0)	21 (5.9)	21 (3.8)	-	0 (0.0)	0 (0.0)
Previous diagnosis of mental health problems	Yes	166 (31.5)	-	77 (22.1)	77 (22.1)	107 (36.6)	11 (6.0)	118 (24.8)
	No	361 (68.5)	-	265 (75.9)	265 (75.9)	182 (62.3)	170 (92.4)	352 (73.9)
	I don’t know	-	-	7 (2.0)	7 (2.0)	3 (1.0)	3 (1.6)	6 (1.3)
	*Missings*	-	-	4 (1.1)	201 (36.5)	0 (0.0)	0 (0.0)	0 (0.0)
Clinically significant symptoms of depression^a^	Yes	-	-	41 (11.9)	41 (11.9)	42 (14.5)	37 (20.1)	79 (16.7)
	No	-	-	304 (88.1)	304 (88.1)	248 (85.5)	147 (79.9)	395 (83.3)
	*Missings*	-	-	8 (2.3)	205 (37.3)	2 (0.7)	0 (0.0)	2 (0.4)
Clinically significant symptoms of anxiety^b^	Yes	-	-	50 (27.0)	50 (27.0)	-	-	-
	No	-	-	135 (73.0)	135 (73.0)	-	-	-
	*Missings*	-	-	168 (47.6)	168 (47.6)	-	-	-
Previous traumatic childbirth	Yes	-	-	35 (24.6)	35 (24.6)	94 (61.8)	23 (30.7)	117 (51.5)
	No	-	-	107 (75.4)	107 (75.4)	58 (38.2)	52 (69.3)	110 (48.5)
	*Missings*	-	-	211 (59.8)	211 (59.8)	140 (47.9)	109 (59.2)	249 (52.3)
Gestational weeks	*M (SD)*	-	29.89(5.89)	33.44 (3.73)	31.74(5.19)	-	-	-
	*Missings*	-	0(0)	137(38.80)	137(38.80)	-	-	-
Gestational weeks at birth	*M (SD)*	-	-	38.31(1.91)	38.31(1.91)	38.52(1.82)	39.36(2.13)	38.84(1.99)
	*Missings*		-	180(50.99)	180(50.99)	3(1.03)	0(0)	3(1.03)

^a^Edinburgh Postnatal Depression Scale total score ≥ 13. ^b^State Anxiety Inventory total score ≥ 45. “-“: data not available

Differences in sociodemographic, obstetric, fetal/neonatal, and mental health-related characteristics by country according to childbearing-age period are depicted in Supplementary Table 2.

### Procedures

Data comes from larger studies conducted in four countries – Australia, Lithuania, Poland, and Portugal (Pinto et al. 2023), which were conducted following the guidelines from the Helsinki Declaration and received approval from the Ethical Commissions of the involved institutions or the Biomedical Research Ethics Committee (Australia: 2022/ETH01110 omitted for blinded review; Lithuania: P1-BE-2-73/2020 and 2024-BEC2-153 omitted for blinded review; Poland: EBEfR20/2022 omitted for blinded review; Portugal: CES CHUSJ: 279 / 2022; MAC:1317/2022; CHPVVC: 10/2022 omitted for blinded review).

In Australia, the sample was recruited in person when women attended the antenatal clinics (January-December 2023), in Poland at the university (November 2023-February 2024), and in Portugal at obstetric units of three large public hospitals (January 2023-February 2024). In Lithuania, recruitment was conducted online (December 2023-February 2024) through social media platforms (e.g., Facebook and Instagram groups for pregnant and postpartum women).

Nulliparous non-pregnant women (Poland), pregnant women (Lithuania, Portugal), and women at two months postpartum (Australia, Lithuania) completed the TSS and self-reported questionnaires to collect sociodemographic, obstetric, fetal/neonatal, and mental health-related data. Additionally, participants from all countries completed self-report measures to assess depressive symptoms and fear of childbirth and participants in Poland and Portugal completed a self-report measure to assess anxiety symptoms (see Supplementary Table 3 for details on measures by country). In Portugal the study is longitudinal, therefore a sub-sample of pregnant women (*n* = 166) also completed information at two months postpartum on type of birth, birth trauma perception (one item from the sociodemographic questionnaire), and depressive and anxiety symptoms (*n* = 166) (see sociodemographic, obstetric, and mental health-related factors in supplementary Table 4).

### Measures

#### Tokophobia Severity Scale

The TSS (Wootton et al. 2020; Martin et al. 2021) is a 13-item self-reported measure scored on a four Likert-type scale from 0 = never to 3 = always). Participants were instructed to indicate the extent to which each item has applied to them over the past 2 weeks (see TSS items in Table 3). Nulliparous non-pregnant women were instructed to complete the TSS by imagining themselves in a pregnancy situation. Women who completed the TSS during pregnancy were instructed to respond based on how they might feel about the current pregnancy or future childbirth. Women who completed the TSS during the postpartum period were instructed to respond based on how they might feel about a future pregnancy or childbirth, thinking about the most recent childbirth experience. TSS total scores range from 0 to 39, with higher scores indicating greater severity of tokophobia symptoms. In each country, the TSS items were translated following best practices and methodological recommendations (Streiner & Kottner 2014). Pilot testing was then conducted with potential end-users. The TSS was developed in Australia, and for the remaining countries, the items were translated following best practices and methodological recommendations (Streiner & Kottner 2014). In short, the translation process included (1) forward translation by two independent translators, (2) synthesis of the translations into a single version, (3) back-translation by a translator blind to the original instrument, and (4) expert panel review to solve inaccuracies, discrepancies and ambiguities.

#### Sociodemographic, obstetric, fetal/neonatal, and mental health-related characteristics

Sociodemographic (age, ethnicity, perception of income, marital status, education), obstetric and fetal/neonatal (parity, previous pregnancy loss, obstetric complications in the current pregnancy, fetal/neonatal complications, type of birth, and gestational age at childbirth), and mental health-related characteristics (previous potentially traumatic lifetime events, previous diagnosis of mental health problems, and previous traumatic childbirth) were collected using a self-reported questionnaire. For nulliparous non-pregnant women, data was collected on childbirth-related characteristics (plans to have children, preferred type of childbirth, perception of childbirth risk, witnessed a traumatic childbirth, being told a traumatic childbirth story).

#### Depressive symptoms

The EPDS (Cox et al. 1987) was used in Australia, Lithuania, and Portugal and the depression subscale of the HADS (Zigmond and Snaith 1983) was used in Poland to assess depressive symptoms. The EPDS is a 10-item self-report measure, scored on a four-point Likert-type scale (ranging from 0 = never to 3 = always). It refers to the occurrence and severity of depressive symptoms in the preceding seven days. Total scores range from 0 to 30, with higher scores indicating higher symptom severity. It showed good internal consistency (over 0.80 as recommended by George and Mallery 2019) for pregnancy and postpartum, in women from the countries included in our study (Australia, Cronbach’s α = 0.81 in postpartum; Lithuania, Cronbach’s α = 0.91 in postpartum; Portugal, Cronbach’s α = 0.82 in pregnancy and 0.88 in postpartum) (Boyce at al. 1993; Lapkienė et al. 2004; Tendais et al. 2014). In our sample, EPDS Cronbach’s α were 0.89 in the Australian sample, 0.87 in the Lithuanian sample, and 0.86 in pregnant women and 0.88 in women in the postpartum period in Portugal.

The depression subscale of the HADS is a 7-item self-report rating scale on depressive symptoms during the past week, scored on a 4-point Likert-type scale (ranging from 0 = not at all to 3 = most of the time). Scores range from 0 to 21, with higher scores indicating higher symptom severity. It showed good internal consistency in women from Poland (Cronbach’s α = 0.78 for depression subscale) (Watrowski and Rohde 2014). In our sample, Cronbach’s α coefficient was 0.73.

#### Anxiety symptoms

The STAI-S (Spielberger et al. 1983) and the anxiety subscale of the HADS (Zigmond and Snaith 1983) were used to assess anxiety symptoms in Portugal and Poland, respectively. The STAI-S is a 20-item self-report measure, scored on a four-point Likert-type scale (ranging from 1 = almost never to 4 = almost always) to assess the occurrence and severity of current anxiety symptoms. Scores range from 20 to 80, with higher scores indicating greater symptom severity. It showed good internal consistency in women from Portugal (Cronbach’s α = 0.91 in pregnancy and 0.92 in postpartum) (Tendais et al. 2014) and in our sample (Cronbach’s α = 0.93 in pregnancy and 0.94 in postpartum).

The anxiety subscale of the HADS is a 7-item self-report rating scale measuring the presence of anxiety symptoms during the past week, scored on a 4-point Likert-type scale (ranging from 0 = not at all to 3 = most of the time). Scores range from 0 to 21, and higher scores indicate greater symptom severity. It showed good internal consistency in women from Poland (Cronbach’s = 0.84 for anxiety subscale) (Watrowski and Rohde 2014). In our sample, Cronbach’s α coefficient was 0.71.

#### Fear of childbirth

The Fear of Birth Scale (FOBS; Hains et al. 2011) is a 2-item self-report questionnaire to assess feelings regarding childbirth. Participants are asked to rate their feelings concerning childbirth by responding to the question “How do you feel right now about the approaching birth?”, by marking an “X” on two 10 cm lines, from 0“no fear” or “calm” and 10 “strong fear” or “worried” (Haines et al. 2011). The instructions were adapted to the respective childbearing period. The total score is derived by calculating the average of the two items, ranging from 0 to 100, and higher scores indicate more fear of childbirth. A cut-off score of FOBS ≥ 50 was used to identify clinically significant symptoms of fear of childbirth, based on previous research with pregnant women (Haines et al. 2011). FOBS’s original version has good internal consistency (Cronbach’s α = .91 in pregnancy; Haines et al. 2011). In our sample, FOBS Cronbach’s α coefficients were .90 in Australia; .93 in pregnant women from Lithuania and .88 in women in the postpartum period from Lithuania; .89 in Poland, and .88 in Portugal.

### Statistical analysis

We first describe participants’ sociodemographic, obstetric, fetal/neonatal, and mental health-related characteristics overall and by country and differences between countries by childbearing-age period (pregnant women, women in the postpartum period) were calculated using chi-square tests (for ordinal variables) and t-tests (for continuous variables).

Analyses of (1) factor structure and measurement invariance, (2) reliability, (3) criterion (known groups and concurrent) validity, (4) convergent validity, (5) predictive validity, and (6) clinical validity were conducted. Significance was considered at *p* < .05. R software (version 4.4.0) with *lavaan* package (version 0.6–16), and *semTools* package (version 0.5-6) were used to perform the exploratory factor analyses (EFA), the confirmatory factor analyses (CFA) and the measurement invariance analyses. IBM SPSS Statistics (version 29) was used to conduct the remaining statistical analyses. Microsoft Excel sheet was used to calculate positive and negative predictive values and accuracy based on sensitivity and specificity values provided by the analysis conducted in SPSS.

#### TSS factor structure

Was initially tested in the total sample of 1,585 women and the model fit was low. Therefore, the sample was randomly divided into two subsamples (subsample 1, *n* = 764; subsample 2, *n* = 821) to conduct an EFA and CFA. EFA was performed with subsample 1, using Unweighted Least Squares Mean, as recommended for ordinal data (Rogers 2021) and varimax rotation. The Bartlett sphericity test and Kaiser–Meyer–Olkin index (KMO) statistics were used to assess model goodness of fit. A significant Bartlett sphericity test and KMO ≥ 0.70 were considered a good fit for the data (Watkins 2018). Visual inspection of the scree plot, Horn’s method of parallel analysis (Horn 1965), and theoretical convergence were used to decide the number of factors to retain, and factor loadings ≥ 0.40 were considered (Howard 2015). The structure obtained in EFA, as well as the structure proposed by Martin et al. (2021), were then examined through CFAs with subsample 2, using Unweighted Least Squares Mean and Variance adjusted estimator, as recommended for ordinal data (Savalei and Rhemtulla 2013): M1: model with three factors and 10 items previously tested by Martin et al. (2021); M2: model with three factors and 13 items given by the EFA analysis with our subsample 1.Since we have floor effects on items 7, 10, and 11 we conducted additional models M1b and M2b: previous models (M1 and M2) without the items with floor effects – items 7, 10 and 11; and found that the model fit did not improve. Therefore, we took into account both the modification indices and semantic similarity of the items and allowed correlation of the residuals for items 5 (“I worry about medical complications during pregnancy and/or childbirth.”) and 9 (“I am concerned that pregnancy and/or childbirth are very painful.”) from the Coping subscale and items 4 (“I am worried that something terrible will happen to my baby during pregnancy and/or delivery.”) and 13 (“I’m worried that something dangerous will happen to my baby during pregnancy or childbirth [e.g. injury or death].”) from the fear subscale. This was model M2c: EFA model with all items and correlated errors on items 5 and 9, and items 4 and 13. The chi-square statistic (χ2), alongside its degrees of freedom, the Comparative Fit Index (CFI), the Tucker-Lewis Index (TLI), the Root Mean Square Error of Approximation (RMSEA 90%CI), and the Standardized Root Mean Squared Residual (SRMR) were used to assess model goodness of fit. Values of $\:{x}^{2}$df ≤ 3, CFI ≥ 0.95, TLI ≥ 0.95, RMSEA ≤ 0.06, and SRMR ≤ 0.08 were considered a good fit to the data, and $\:{x}^{2}$/df < 5, CFI ≥ 0.90, TLI ≥ 0.90, RMSEA ≤ 0.08, and SRMR ≤. 08 were considered an acceptable fit to the data (Kline 2023).

Measurement invariance was assessed through multi-group analysis with the total sample. Before testing for measurement invariance, the CFA model’s goodness of fit was tested separately for each childbearing-age period (nulliparous non-pregnant women, *n* = 559; pregnant women, *n* = 550; and women in the postpartum period, *n* = 476) and then within each childbearing-age period for each country (except for nulliparous non-pregnant women, for which we only have data for one country). Since the models demonstrated an acceptable fit in all groups, measurement invariance across childbearing-age periods was examined. Configural invariance was assessed through CFA model goodness-of-fit indices, including all childbearing-age period. Since data from pregnant women and women in the postpartum period comes from more than one country, measurement invariance by country for each childbearing-age period was also analyzed. In this case, configural invariance was assessed through CFA model goodness-of-fit indices, including all countries within childbearing-age period (Lithuania, *n* = 197; and Portugal for pregnant women, *n* = 353; Australia, *n* = 292 and Lithuania, *n* = 184 for women in the postpartum period). Since configural invariance was always confirmed, in the next step increasing constraints were imposed on thresholds as recommended for ordinal data (Bowen and Masa 2015) and finally increasing constraints on thresholds plus factor loadings to test scalar invariance as recommended (scalar invariance; Svetina et al. 2019; Wu and Estabrook 2016). At each level, constrained and free models were compared using the chi-square difference test (Satorra and Bentler 2001), ΔCFI, and ΔRMSEA. Lack of invariance was considered if significant chi-square difference tests, ΔCFI ≤ − 0.002, and ΔRMSEA ≥ 0.010 (Rutkowski and Svetina 2017). Since none of the women in the postpartum from Lithuania provided responses on category 3 of item 11 (range 0–2), categories 2 and 3 were merged (as proposed by Svetina et al. 2019) to allow the evaluation of measurement invariance by country for postpartum data.

#### Reliability

Was examined using Cronbach’s alpha coefficient, McDonald’s Omega coefficient (ω), mean inter-item correlations, and corrected item-total correlations (Spearman correlations were used due to the ordinal nature of the items).

McDonald’s Omega coefficient (ω) was calculated since Cronbach’s alpha requires several assumptions, namely equal factor loadings across all items (Flora 2020; Savalei and Reise 2019). Cronbach’s alpha or McDonald’s Omega coefficient values ≥ 0.70 were considered acceptable, ≥ 0.80 were considered good, and ≥ 0.90 were considered excellent (George and Mallery 2019). Mean inter-item correlation between 0.20 and 0.40 and corrected item-total correlations higher than 0.30 were considered adequate (Piedmont 2014; Vaus 2002).

#### Criterion validity (known groups and concurrent) were examined by testing

##### Known-groups validity

Differences in TSS total scores in nulliparous non-pregnant women, pregnant women, and women in the postpartum period according to sociodemographic, obstetric, fetal/neonatal, and mental health-related characteristics were assessed using two-way analysis of variance (ANOVAs). When data were available for only one country, independent sample *t*-tests or one-way ANOVA were conducted. Assumptions of normality and homogeneity of variances were tested. Non-parametric tests (e.g., non-parametric two-way ANOVA, Mann-Whitney, and Kruskal-Wallis) were performed when the assumptions were not met. Results from parametric tests were reported when parametric and non-parametric tests provided the same results. For all ANOVAs, Tukey HSD correction was used for post-hoc tests. Effect sizes (partial eta squared and Cohen’s *d*) were calculated. According to Cohen (1988), partial eta squared values of 0.01, 0.06, and 0.14 represent small, medium, and large effects, respectively, and *d* = 0.2, *d* = 0.5, and *d* = 0.8 represent small, moderate, and large effects, respectively.

##### Concurrent validity

was examined through Pearson correlations between TSS, depressive and anxiety symptoms, and birth trauma perception. Correlation results were interpreted according to Cohen’s guidelines (Cohen 1988): values between 0.10 and 0.29, between 0.30 and 0.49, and between 0.50 and 1.00 correspond to weak, moderate, and strong size correlations, respectively.

### Convergent validity

 Was examined through Pearson correlations between TSS and fear of childbirth (FOBS)

#### Predictive validity

Was examined using longitudinal data from Portugal (subsample *n* = 166) by testing TSS scores in pregnant women as a predictor of the type of birth (elective cesarean section vs. other [vaginal, assisted vaginal, or emergency cesarean section]) using logistic regression, and of birth trauma perception, and postpartum depressive and anxiety symptoms, using linear regressions (birth trauma perception, and postpartum depressive and anxiety symptoms). For linear regressions, the assumptions of linearity, normal distribution, and homogeneity of errors were validated through residual plots. The Durbin-Watson statistic validated the independence of errors assumption (d ≈ 2; ±0.4). Due to high correlations between all TSS subscales, four linear regressions were performed for each independent variable (one for each TSS subscale and one for TSS total score as independent variables) to avoid multicollinearity issues.

##### Clinical validity

was examined using Receiver Operating Curve (ROC) analysis with FOBS. The screening performance of the TSS to identify women with (FOBS ≥ 50) and without (FOBS < 50) clinically significant tokophobia symptoms was assessed using the area under the curve (AUC; 95% CI). AUC ≥ 0.70, ≥ 0.80, and ≥ 0.90 were indicative of acceptable, good, and exceptional predictive ability, respectively (Hosmer and Lemeshow 2000). Sensitivity, specificity, positive and negative predictive values, and accuracy were calculated for TSS cutoff scores. Sensitivity and specificity between 50% and 80% or higher than 80% were indicative of reasonable and good predictive ability, respectively (Hosmer and Lemeshow 2000). The optimal cutoff score was determined by identifying the value closer to the intersection between the ROC curve and the diagonal line from the upper left corner to the lower right corner of the graph (Bland 2000).

## Results

No differences between countries were found in TSS total scores in pregnant women (*t* (548) = -1.00; *p* = .318; *d* = 0.09) or women in the postpartum period (*t* (474) = 0.28; *p* = .404; *d* = 0.03). Supplementary Table 5 shows descriptive statistics EPDS, STAI-S, and FOBS.

### Factor structure and measurement invariance

#### Factor structure

The model proposed by Martin et al. (2021), with three factors and 10 items, did not have an acceptable fit in our total sample (model M1). Items with floor effects were removed (items 7, 10, and 11) but the fit became worse (model M1b), thus all items were retained (see Supplementary Table 6).

The Bartlett sphericity test (χ2 (78) = 5887.63; *p* < .001) and KMO = 0.89 suggested that the data from subsample 1 was adequate for EFA. Three factors were extracted. Items loading in more than one factor (items 1, 2, 6, and 8) were included in the factor with higher loading weight (items 1, 2, and 6), except item 8 which was included in factor 2 due to semantic and conceptual meaning content. Items 1, 3, 4, 12, and 13 loaded in Factor 1 (Fear); items 2, 5, 6, 8, and 9 loaded in Factor 2 (Coping); and items 7, 10, and 11 loaded in Factor 3 (Intrusive thoughts) (see Table 2).

**Table 2:** Tokophobia Severity Scale factor structure: factor loadings, communalities and percentage of variance explained from the exploratory factor analysis with varimax rotation (*N* = 764)

Item	Factor Loadings			h^2^
	Factor 1 (Fear)	Factor 2 (Coping)	Factor 3 (Intrusive thoughts)	
Item 1: I worry about medical complications during pregnancy and/or childbirth	0.63	0.40	0.19	0.59
Item 2: I worry about the type of delivery that I will have when I have a baby	0.49	0.51	0.21	0.55
Item 3: I worry that something terrible will happen to me during my pregnancy and/or childbirth	0.73	0.35	0.24	0.71
Item 4: I worry that something terrible will happen to my baby during my pregnancy and/or childbirth	0.83	0.17	0.12	0.73
Item 5: I worry that I will not be able to cope with the pain of pregnancy and/or childbirth	0.26	0.80	0.21	0.75
Item 6: I worry about the medical procedures required during pregnancy and/or childbirth	0.46	0.49	0.24	0.50
Item 7: I avoid talking about pregnancy, children and childbirth because of my fears	0.17	0.17	0.50	0.31
Item 8: I worry that I will not be in control of the medical procedures during my pregnancy and/or delivery	0.46	0.40	0.34	0.49
Item 9: I worry that pregnancy and/or childbirth will be too painful	0.22	0.82	0.21	0.77
Item 10: I check excessively to determine if I am pregnant	0.20	0.15	0.49	0.30
Item 11: I have nightmares about being pregnant and/or delivering a child	0.09	0.13	0.82	0.70
Item 12: I worry that something dangerous will happen to me during pregnancy or the delivery (e.g., a ruptured uterus, preeclampsia, emergency interventions, death)	0.65	0.33	0.33	0.65
Item 13: I worry that something dangerous will happen to my child during pregnancy or the delivery (e.g., injury or death)	0.82	0.15	0.18	0.72
% variance explained	27	19	13	-

h^2^ = communalities. Factor loadings equal to or higher than 0.40 are in bold. Data from subsample 1 (*n* = 764)

The results of the CFA (conducted with subsample 2) of the 3-factor model given by the EFA (M2) showed an acceptable fit in most fit indices. Deleting items with floor effects (items 7, 10, and 11) did not improve the model fit (M2b). The model M2c with correlated residuals for items 5 and 9, and items 4 and 13 were good according to all fit measures (see Table 3).

**Table 3:** Tokophobia Severity Scale factor structure: confirmatory factor analyses of different models (*N* = 821)

Model	$\:{{\upchi\:}}^{2}$	* df*	*p*	$\:{{\upchi\:}}^{2}/df$	RMSEA	90% CI RMSEA	SRMR	CFI	TLI
M1	495.02	32	< 0.001	15.47	0.13	0.12-0.14	0.07	0.94	0.91
M1b	730.62	19	< 0.001	38.45	0.21	0.20-0.23	0.08	0.91	0.86
M2	392.47	62	< 0.001	6.33	0.08	0.07-0.09	0.05	0.97	0.96
M2b	545.49	34	< 0.001	16.04	0.14	0.13-0.15	0.06	0.95	0.94
M2c	176.13	60	< 0.001	2.94	0.05	0.04-0.06	0.03	0.99	0.98

M1: model with three factors and 10 items proposed by Martin et al. (2022); M2: model with three factors and 13 items given by the EFA analysis with our subsample 1; M1b and M2b: previous models without the items with floor effects – items 7, 10 and 11; M2c: EFA model with correlated errors on items 5 and 9, and items 4 and 13. The confirmatory factor analyses were performed with data from subsample 2, with *n* = 821. χ^2^ = chi-square statistic; *df =* degree of freedom; *p = p-value; RMSEA =* Root Mean Square Error of Approximation; SRMR = Standardized Root Mean Squared Residual; CFI = Comparative Fit Index; TLI = Tucker-Lewis Index

**Fig. 1:**
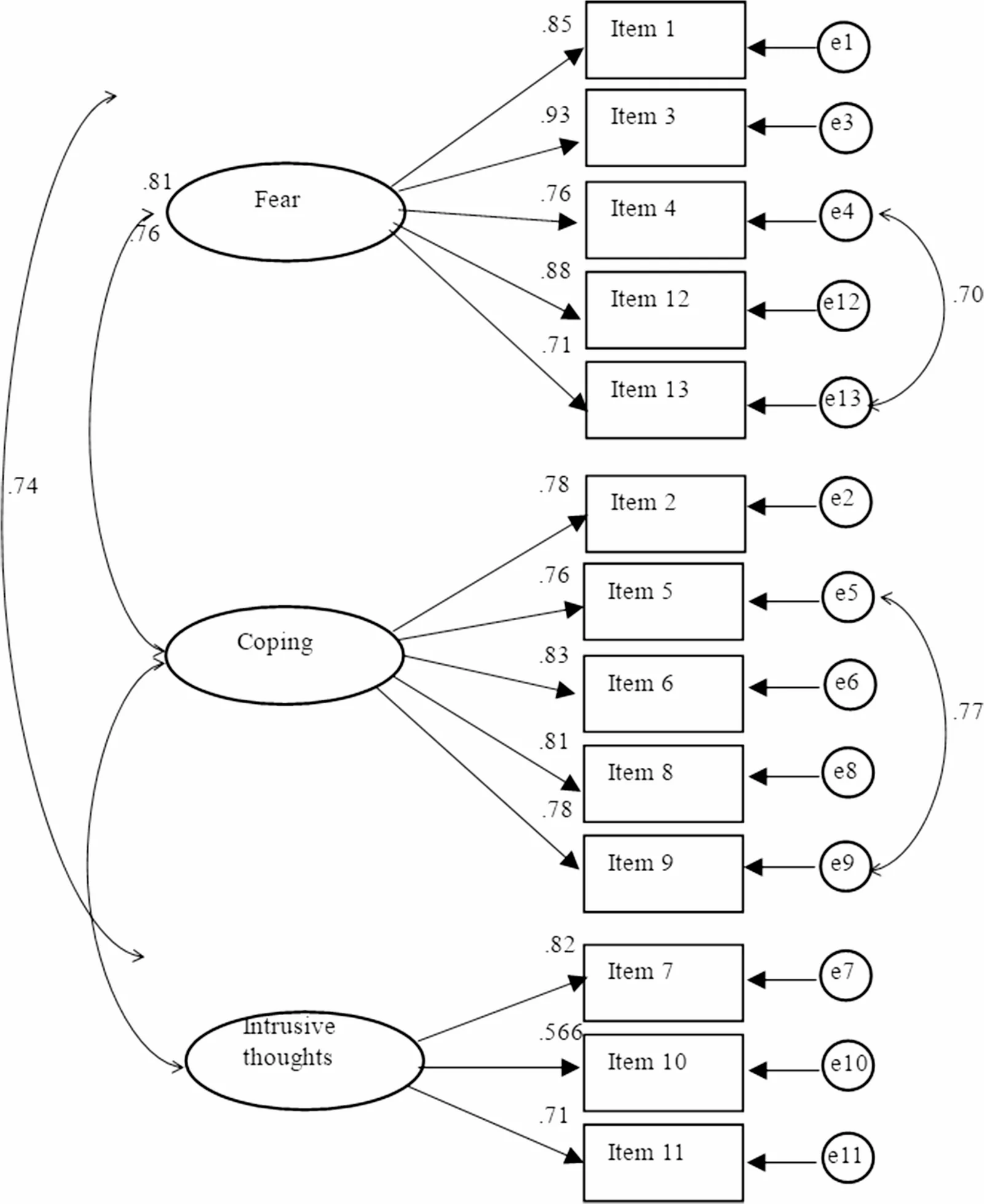
Tokophobia Severity Scale factor structure: model with 3 factors, 13 items, correlated errors and standardized factor loadings (*N* = 821)

The 3-factor model proposed by Martin et al. (2021) with and without items with floor effects (M1 and M1b) also did not show an acceptable fit in subsample 2. Therefore, the 3-factor model given by the EFA with correlated errors was selected (M2c; see Table 3; Fig. 1).

Item 1: I worry about medical complications during pregnancy and/or childbirth; Item 2: I worry about the type of delivery that I will have when I have a baby; Item 3: I worry that something terrible will happen to me during my pregnancy and/or childbirth; Item 4: I worry that something terrible will happen to my baby during my pregnancy and/or childbirth; Item 5: I worry that I will not be able to cope with the pain of pregnancy and/or childbirth; Item 6: I worry about the medical procedures required during pregnancy and/or childbirth; Item 7: I avoid talking about pregnancy, children and childbirth because of my fears; Item 8: I worry that I will not be in control of the medical procedures during my pregnancy and/or delivery; Item 9: I worry that pregnancy and/or childbirth will be too painful; Item 10: I check excessively to determine if I am pregnant; Item 11: I have nightmares about being pregnant and/or delivering a child; Item 12: I worry that something dangerous will happen to me during pregnancy or the delivery (e.g., a ruptured uterus, preeclampsia, emergency interventions, death); Item 13: I worry that something dangerous will happen to my child during pregnancy or the delivery (e.g., injury or death).

#### Measurement invariance

The configural model has an acceptable fit. Furthermore, the Δ**χ2**significance analysis, ΔRMSEA, and ΔCFI values were indicative of thresholds and loadings invariance between childbearing-age periods and between countries (See Table 4).

**Table 4:** Tokophobia Severity Scale measurement invariance: model comparisons by childbearing-age period and country (*N* = 1585)

Model	$\:{{\upchi\:}}^{2}$	df	*p*	$\:{{\upchi\:}}^{2}/df$	RMSEA	90% CI RMSEA	SRMR	CFI	TLI	Scaled chi-square difference test^a^			ΔRMSEA	ΔCFI
										${{\Delta\:}}$ ${{{\upchi\:}}^{2}}_{scaled}$	$\:{\Delta\:}df$	*p*		
Comparisons by childbearing-age period														
Nulliparous non-pregnant women (*n* = 559)^1^	192.73	60	< 0.001	3.21	0.06	0.05-0.07	0.05	0.98	0.98	-	-	-	-	-
Pregnant women (*n* = 550)^2^	170.93	60	< 0.001	2.85	0.06	0.05-0.07	0.05	0.97	0.98	-	-	-	-	-
Postpartum women (*n* = 476)^3^	125.42	60	< 0.001	2.09	0.05	0.04-0.06	0.05	0.98	0.97	-	-	-	-	-
Configural	475.39	180	< 0.001	2.64	0.06	0.05-0.06	0.05	0.98	0.98	-	-	-	-	-
Thresholds	506.35	206	< 0.001	2.46	0.05	0.05-0.06	0.05	0.98	0.97	52.77	26	0.001	− 0.003	0.000
Thresholds + Loadings (scalar invariance)	549.81	226	< 0.001	2.43	0.05	0.05-0.06	0.05	0.97	0.97	66.47	20	< 0.001	0.000	− 0.002
Comparisons by country														
Pregnant women (*n* = 550)														
Lithuania (*n* = 197)	120.24	60	< 0.001	2.00	0.07	0.05-0.09	0.07	0.96	0.95	-	-	-	-	-
Portugal (*n* = 353)	124.39	60	< 0.001	2.07	0.06	0.04-0.07	0.05	0.98	0.97	-	-	-	-	-
Configural	244.51	120	< 0.001	2.04	0.06	0.05-0.07	0.06	0.97	0.96	-	-	-	-	-
Thresholds	232.20	133	< 0.001	1.75	0.05	0.04-0.06	0.06	0.98	0.97	11.41	13	0.577	− 0.009	0.006
Thresholds + Loadings (scalar invariance)	238.24	143	< 0.001	1.67	0.05	0.04-0.06	0.06	0.98	0.98	18.06	10	0.054	− 0.003	0.001
Postpartum women (*n* = 476)														
Australia (*n* = 292)	98.30	60	0.001	1.64	0.05	0.03-0.06	0.05	0.98	0.98	-	-	-	-	-
Lithuania (*n* = 184)	83.88	60	0.023	1.40	0.05	0.02.07	0.06	0.98	0.98	-	-	-	-	-
Configural	179.12	120	< 0.001	1.49	0.05	0.03-0.06	0.06	0.98	0.98	-	-	-	-	-
Thresholds	195.75	132	< 0.001	1.48	0.05	0.03-0.06	0.06	0.98	0.98	18.15	12	0.111	0.000	− 0.001
Thresholds + Loadings (scalar invariance)	186.45	142	< 0.001	1.31	0.04	0.02-0.05	0.06	0.99	0.99	8.74	10	0.557	− 0.009	0.006

^*1,2 e 3*^Before testing for measurement invariance, the model’s goodness of fit was evaluated separately for each group (nulliparous non-pregnant women, pregnant women, and women in the postpartum period). Since the model demonstrated an acceptable fit in all groups, measurement invariance across childbearing-age periods was examined.^*a*^$\:{\Delta\:}{{\upchi\:}}^{2}$was calculated using Satorra and Bentler (2001) proposed scaled chi-square difference test; χ^2^  = chi-square statistic; *df*  degree of freedom, *p = p-value; RMSEA =* Root Mean Square Error of Approximation; *SRMR*  Standardized Root Mean Squared Residual, *CFI*  Comparative Fit Index, *TLI*  Tucker-Lewis Index

### Reliability

Cronbach’s alpha and McDonald’s omega coefficient for total score and the Fear and Coping subscales ranged from 0.83 to 0.89, and for the intrusive thoughts subscale, ranged from 0.46 to 0.65. Mean inter-item correlations ranged between 0.33 and 0.40. Items 10 and 11 had corrected item-total correlations lower than 0.30 in some countries and items 7, 10 and 11 had floor effects in all countries (see Table 5).

**Table 5:** Tokophobia Severity Scale internal consistency: reliability and descriptive statistics by country and childbearing-age period (*N* = 1585)

TSS	α	α IID	ω	ω IID	MIC	cITC	M	SD	Range	P25	P50	P75
**Poland (**nulliparous non-pregnant women; ***n*** **= 559)**												
Item 1 - I worry about medical complications during pregnancy and/or childbirth		0.89		0.88		0.71	2.03	0.88	0–3	1.00	2.00	3.00
Item 2 - I worry about the type of delivery that I will have when I have a baby		0.89		0.89		0.68	1.66	0.98	0–3	1.00	2.00	2.00
Item 3 - I worry that something terrible will happen to me during my pregnancy and/or childbirth		0.88		0.88		0.77	1.87	0.96	0–3	1.00	2.00	3.00
Item 4 - I worry that something terrible will happen to my baby during my pregnancy and/or childbirth		0.89		0.89		0.57	1.88	0.92	0–3	1.00	2.00	3.00
Item 5 - I worry that I will not be able to cope with the pain of pregnancy and/or childbirth		0.89		0.89		0.63	1.88	1.02	0–3	1.00	2.00	3.00
Item 6 - I worry about the medical procedures required during pregnancy and/or childbirth		0.89		0.89		0.64	1.60	1.02	0–3	1.00	2.00	2.00
Item 7 - I avoid talking about pregnancy, children and childbirth because of my fears		0.90		0.90		0.42	0.55	0.84	0–3	0.00	0.00	1.00
Item 8 - I worry that I will not be in control of the medical procedures during my pregnancy and/or delivery		0.89		0.89		0.64	1.59	0.98	0–3	1.00	2.00	2.00
Item 9 - I worry that pregnancy and/or childbirth will be too painful		0.89		0.89		0.67	1.96	1.01	0–3	1.00	2.00	3.00
Item 10 - I check excessively to determine if I am pregnant		0.90		0.90		0.33	0.84	1.01	0–3	0.00	0.00	1.00
Item 11 - I have nightmares about being pregnant and/or delivering a child		0.90		0.90		0.41	0.59	0.90	0–3	0.00	0.00	1.00
Item 12 - I worry that something dangerous will happen to me during pregnancy or the delivery (e.g., a ruptured uterus, preeclampsia, emergency interventions, death)		0.88		0.88		0.77	1.77	1.02	0–3	1.00	2.00	3.00
Item 13 - I worry that something dangerous will happen to my child during pregnancy or the delivery (e.g., injury or death)		0.89		0.89		0.59	1.71	0.96	0–3	1.00	2.00	3.00
TSS total score	0.90		0.90		0.40		19.93	8.41	0–39	13.00	20.00	27.00
TSS fear subscale score	0.88		0.88		0.60		9.27	3.92	0–15	6.00	9.00	13.00
TSS coping subscale score	0.84		0.84		0.52		8.69	3.95	0–15	6.00	9.00	12.00
TSS Intrusive thoughts subscale score	0.65		0.68		0.37		1.98	2.11	0–9	0.00	1.00	3.00
Lithuania (Pregnant women; *n* = 197)												
Item 1 - I worry about medical complications during pregnancy and/or childbirth		0.89		0.89		0.67	1.30	0.79	0–3	1.00	1.00	2.00
Item 2 - I worry about the type of delivery that I will have when I have a baby		0.89		0.89		0.63	1.13	0.85	0–3	1.00	1.00	2.00
Item 3 - I worry that something terrible will happen to me during my pregnancy and/or childbirth		0.89		0.89		0.71	1.17	0.86	0–3	1.00	1.00	2.00
Item 4 - I worry that something terrible will happen to my baby during my pregnancy and/or childbirth		0.89		0.89		0.59	1.16	0.82	0–3	1.00	1.00	1.50
Item 5 - I worry that I will not be able to cope with the pain of pregnancy and/or childbirth		0.89		0.89		0.65	1.15	0.93	0–3	0.00	1.00	2.00
Item 6 - I worry about the medical procedures required during pregnancy and/or childbirth		0.89		0.89		0.64	1.10	0.89	0–3	0.00	1.00	2.00
Item 7 - I avoid talking about pregnancy, children and childbirth because of my fears		0.90		0.90		0.45	0.38	0.72	0–3	0.00	0.00	1.00
Item 8 - I worry that I will not be in control of the medical procedures during my pregnancy and/or delivery		0.89		0.89		0.54	0.95	0.99	0–3	0.00	1.00	2.00
Item 9 - I worry that pregnancy and/or childbirth will be too painful		0.89		0.89		0.65	1.19	0.95	0–3	0.00	1.00	2.00
Item 10 - I check excessively to determine if I am pregnant		0.90		0.90		0.24	0.27	0.56	0–3	0.00	0.00	0.00
Item 11 - I have nightmares about being pregnant and/or delivering a child		0.90		0.90		0.38	0.28	0.55	0–3	0.00	0.00	0.00
Item 12 - I worry that something dangerous will happen to me during pregnancy or the delivery (e.g., a ruptured uterus, preeclampsia, emergency interventions, death)		0.89		0.89		0.55	0.88	0.80	0–3	0.00	1.00	1.00
Item 13 - I worry that something dangerous will happen to my child during pregnancy or the delivery (e.g., injury or death)		0.89		0.90		0.51	1.02	0.80	0–3	1.00	1.00	1.00
TSS total score	0.90		0.90		0.36		11.97	7.16	0–39	7.00	11.00	15.50
TSS fear subscale score	0.88		0.88		0.55		5.52	3.33	0–15	4.00	5.00	7.00
TSS coping subscale score	0.84		0.87		0.56		5.52	3.78	0–15	2.50	5.00	8.00
TSS Intrusive thoughts subscale score	0.53		0.53		0.25		0.93	1.33	0–9	0.00	0.00	1.50
Portugal (Pregnant women; *n =* 353)												
Item 1 - I worry about medical complications during pregnancy and/or childbirth		0.89		0.89		0.62	1.40	0.71	0–3	1.00	1.00	2.00
Item 2 - I worry about the type of delivery that I will have when I have a baby		0.88		0.89		0.64	1.29	0.86	0–3	1.00	1.00	2.00
Item 3 - I worry that something terrible will happen to me during my pregnancy and/or childbirth		0.88		0.89		67	1.17	0.86	0–3	1.00	1.00	2.00
Item 4 - I worry that something terrible will happen to my baby during my pregnancy and/or childbirth		0.88		0.89		0.69	1.47	0.88	0–3	1.00	1.00	2.00
Item 5 - I worry that I will not be able to cope with the pain of pregnancy and/or childbirth		0.89		0.89		0.55	1.00	0.84	0–3	0.00	1.00	1.00
Item 6 - I worry about the medical procedures required during pregnancy and/or childbirth		0.89		0.89		0.59	1.13	0.79	0–3	1.00	1.00	2.00
Item 7 - I avoid talking about pregnancy, children and childbirth because of my fears		0.90		0.90		0.33	0.17	0.45	0–3	0.00	0.00	0.00
Item 8 - I worry that I will not be in control of the medical procedures during my pregnancy and/or delivery		0.89		0.89		0.52	0.85	0.82	0–3	0.00	1.00	1.00
Item 9 - I worry that pregnancy and/or childbirth will be too painful		0.89		0.89		0.58	1.06	0.80	0–3	1.00	1.00	1.00
Item 10 - I check excessively to determine if I am pregnant		0.90		0.90		0.34	0.26	0.60	0–3	0.00	0.00	0.00
Item 11 - I have nightmares about being pregnant and/or delivering a child		0.90		0.90		0.26	0.20	0.49	0–3	0.00	0.00	0.00
Item 12 - I worry that something dangerous will happen to me during pregnancy or the delivery (e.g., a ruptured uterus, preeclampsia, emergency interventions, death)		0.88		0.89		0.67	1.17	0.81	0–3	1.00	1.00	2.00
Item 13 - I worry that something dangerous will happen to my child during pregnancy or the delivery (e.g., injury or death)		0.88		0.89		0.71	1.42	0.87	0–3	1.00	1.00	2.00
TSS total score	0.90		0.90		0.37		12.58	6.62	0–34	8.00	11.00	16.00
TSS fear subscale score	0.90		0.91		0.63		6.62	3.50	0–15	4.00	6.00	9.00
TSS coping subscale score	0.84		0.84		0.50		5.33	3.22	0–15	3.00	5.00	7.00
TSS Intrusive thoughts subscale score	0.58		0.61		0.11		0.63	1.15	0–6	0.00	0.00	1.00
Total (Pregnant women; *n* = 550)												
Item 1 - I worry about medical complications during pregnancy and/or childbirth		0.88		0.89		0.64	1.37	0.74	0–3	1.00	1.00	2.00
Item 2 - I worry about the type of delivery that I will have when I have a baby		0.88		0.88		0.63	1.23	0.86	0–3	1.00	1.00	2.00
Item 3 - I worry that something terrible will happen to me during my pregnancy and/or childbirth		0.88		0.88		0.69	1.17	0.86	0–3	1.00	1.00	2.00
Item 4 - I worry that something terrible will happen to my baby during my pregnancy and/or childbirth		0.88		0.89		0.64	1.36	0.87	0–3	1.00	1.00	2.00
Item 5 - I worry that I will not be able to cope with the pain of pregnancy and/or childbirth		0.89		0.89		0.58	1.05	0.87	0–3	0.00	1.00	2.00
Item 6 - I worry about the medical procedures required during pregnancy and/or childbirth		0.88		0.89		0.61	1.12	0.83	0–3	1.00	1.00	2.00
Item 7 - I avoid talking about pregnancy, children and childbirth because of my fears		0.89		0.89		0.36	0.24	0.57	0–3	0.00	0.00	0.00
Item 8 - I worry that I will not be in control of the medical procedures during my pregnancy and/or delivery		0.89		0.89		0.52	0.88	0.88	0–3	0.00	1.00	1.00
Item 9 - I worry that pregnancy and/or childbirth will be too painful		0.89		0.89		0.60	1.11	0.86	0–3	1.00	1.00	2.00
Item 10 - I check excessively to determine if I am pregnant		0.90		90		0.30	0.27	0.59	0–3	0.00	0.00	0.00
Item 11 - I have nightmares about being pregnant and/or delivering a child		0.90		0.89		0.30	0.23	0.51	0–3	0.00	0.00	0.00
Item 12 - I worry that something dangerous will happen to me during pregnancy or the delivery (e.g., a ruptured uterus, preeclampsia, emergency interventions, death)		0.88		0.89		0.62	1.07	0.82	0–3	1.00	1.00	1.00
Item 13 - I worry that something dangerous will happen to my child during pregnancy or the delivery (e.g., injury or death)		0.89		0.89		0.62	1.27	0.87	0–3	1.00	1.00	2.00
TSS total score	0.90		0.90		0.36		12.36	6.82	0–39	8.00	11.00	16.00
TSS fear subscale score	0.89		0.90		0.60		6.23	3.48	0–15	4.00	5.00	8.00
TSS coping subscale score	0.85		0.85		0.52		5.40	3.43	0–15	3.00	5.00	7.00
TSS Intrusive thoughts subscale score	0.56		0.57		0.29		0.74	1.22	0–9	0.00	0.00	1.00
Australia (Postpartum women; *n =* 292)												
Item 1 - I worry about medical complications during pregnancy and/or childbirth		0.88		0.88		0.62	1.35	0.94	0–3	1.00	1.00	2.00
Item 2 - I worry about the type of delivery that I will have when I have a baby		0.88		0.88		0.62	1.15	0.96	0–3	0.00	1.00	2.00
Item 3 - I worry that something terrible will happen to me during my pregnancy and/or childbirth		0.87		0.87		0.71	1.03	0.93	0–3	0.00	1.00	1.00
Item 4 - I worry that something terrible will happen to my baby during my pregnancy and/or childbirth		0.87		0.88		0.70	1.21	0.93	0–3	1.00	1.00	2.00
Item 5 - I worry that I will not be able to cope with the pain of pregnancy and/or childbirth		0.88		0.89		0.59	0.97	0.94	0–3	0.00	1.00	1.00
Item 6 - I worry about the medical procedures required during pregnancy and/or childbirth		0.88		0.88		0.67	1.02	0.87	0–3	0.00	1.00	1.00
Item 7 - I avoid talking about pregnancy, children and childbirth because of my fears		0.89		0.89		0.42	0.24	0.63	0–3	0.00	0.00	0.00
Item 8 - I worry that I will not be in control of the medical procedures during my pregnancy and/or delivery		0.88		0.88		0.55	0.78	0.95	0–3	0.00	1.00	1.00
Item 9 - I worry that pregnancy and/or childbirth will be too painful		0.89		0.89		0.46	0.97	0.98	0–3	0.00	1.00	2.00
Item 10 - I check excessively to determine if I am pregnant		0.89		0.89		0.24	0.33	0.71	0–3	0.00	0.00	0.00
Item 11 - I have nightmares about being pregnant and/or delivering a child		0.89		0.89		0.24	0.19	0.56	0–3	0.00	0.00	0.00
Item 12 - I worry that something dangerous will happen to me during pregnancy or the delivery (e.g., a ruptured uterus, preeclampsia, emergency interventions, death)		0.88		0.88		0.66	1.02	0.95	0–3	0.00	1.00	1.00
Item 13 - I worry that something dangerous will happen to my child during pregnancy or the delivery (e.g., injury or death)		0.88		0.88		0.65	1.08	0.93	0–3	0.00	1.00	1.00
TSS total score	0.89		0.89		0.35		11.33	7.49	0–39	6.00	10.00	15.75
TSS fear subscale score		0.90		0.90	0.61		5.70	3.94	0–15	3.00	5.00	8.00
TSS coping subscale score		0.84		0.82	0.50		4.88	3.66	0–15	2.00	4.00	7.00
TSS Intrusive thoughts subscale score		0.50		0.52	0.24		0.76	1.35	0–9	0.00	0.00	1.00
Lithuania (Postpartum women; *n* = 184)												
Item 1 - I worry about medical complications during pregnancy and/or childbirth		0.88		0.88		0.60	1.22	0.80	0–3	1.00	1.00	2.00
Item 2 - I worry about the type of delivery that I will have when I have a baby		0.88		0.88		0.57	0.89	0.94	0–3	0.00	1.00	1.00
Item 3 - I worry that something terrible will happen to me during my pregnancy and/or childbirth		0.87		0.88		0.67	1.14	0.85	0–3	1.00	1.00	2.00
Item 4 - I worry that something terrible will happen to my baby during my pregnancy and/or childbirth		0.87		0.88		0.63	1.21	0.83	0–3	1.00	1.00	2.00
Item 5 - I worry that I will not be able to cope with the pain of pregnancy and/or childbirth		0.87		0.88		0.64	1.16	1.03	0–3	0.00	1.00	2.00
Item 6 - I worry about the medical procedures required during pregnancy and/or childbirth		0.87		0.88		0.63	0.88	0.91	0–3	0.00	1.00	1.00
Item 7 - I avoid talking about pregnancy, children and childbirth because of my fears		0.89		0.89		0.33	0.27	0.61	0–3	0.00	0.00	0.00
Item 8 - I worry that I will not be in control of the medical procedures during my pregnancy and/or delivery		0.87		0.88		0.61	0.60	0.81	0–3	0.00	0.00	1.00
Item 9 - I worry that pregnancy and/or childbirth will be too painful		0.87		0.88		0.61	1.23	0.94	0–3	1.00	1.00	2.00
Item 10 - I check excessively to determine if I am pregnant		0.90		0.90		0.11	0.37	0.67	0–3	0.00	0.00	1.00
Item 11 - I have nightmares about being pregnant and/or delivering a child		0.89		0.89		0.40	0.29	0.53	0–2	0.00	0.00	0.75
Item 12 - I worry that something dangerous will happen to me during pregnancy or the delivery (e.g., a ruptured uterus, preeclampsia, emergency interventions, death)		0.88		0.88		0.53	0.83	0.81	0–3	0.00	1.00	1.00
Item 13 - I worry that something dangerous will happen to my child during pregnancy or the delivery (e.g., injury or death)		0.88		0.88		0.57	1.07	0.89	0–3	0.00	1.00	1.00
TSS total score	0.89		0.89		0.33		11.14	6.99	0–32	6.00	10.00	15.00
TSS fear subscale score		0.87		0.86	0.52		5.46	3.33	0–15	3.00	5.00	7.75
TSS coping subscale score		0.87		0.86	0.53		4.76	3.73	0–15	2.00	4.00	7.00
TSS Intrusive thoughts subscale score		0.38		0.38	0.19		0.92	1.22	0–5	0.00	0.00	2.00
Total (Postpartum women; *n* = 476)												
Item 1 - I worry about medical complications during pregnancy and/or childbirth		0.88		0.88		0.61	1.30	0.89	0–3	1.00	1.00	2.00
Item 2 - I worry about the type of delivery that I will have when I have a baby		0.88		0.88		0.59	1.05	0.96	0–3	0.00	1.00	2.00
Item 3 - I worry that something terrible will happen to me during my pregnancy and/or childbirth		0.87		0.87		0.69	1.07	0.90	0–3	0.00	1.00	1.00
Item 4 - I worry that something terrible will happen to my baby during my pregnancy and/or childbirth		0.87		0.88		0.67	1.21	0.89	0–3	1.00	1.00	2.00
Item 5 - I worry that I will not be able to cope with the pain of pregnancy and/or childbirth		0.88		0.88		0.61	1.04	0.98	0–3	0.00	1.00	2.00
Item 6 - I worry about the medical procedures required during pregnancy and/or childbirth		0.87		0.88		0.66	0.97	0.89	0–3	0.00	1.00	1.00
Item 7 - I avoid talking about pregnancy, children and childbirth because of my fears		0.88		0.89		0.38	0.25	0.62	0–3	0.00	0.00	0.00
Item 8 - I worry that I will not be in control of the medical procedures during my pregnancy and/or delivery		0.88		0.88		0.57	0.71	0.90	0–3	0.00	0.00	1.00
Item 9 - I worry that pregnancy and/or childbirth will be too painful		0.88		0.89		0.50	1.07	0.97	0–3	0.00	1.00	2.00
Item 10 - I check excessively to determine if I am pregnant		0.89		0.89		0.19	0.34	0.70	0–3	0.00	0.00	0.00
Item 11 - I have nightmares about being pregnant and/or delivering a child		0.89		0.89		0.30	0.23	0.55	0–3	0.00	0.00	0.00
Item 12 - I worry that something dangerous will happen to me during pregnancy or the delivery (e.g., a ruptured uterus, preeclampsia, emergency interventions, death)		0.88		0.88		0.61	0.95	0.91	0–3	0.00	1.00	1.00
Item 13 - I worry that something dangerous will happen to my child during pregnancy or the delivery (e.g., injury or death)		0.87		0.88		0.62	1.07	0.91	0–3	0.00	1.00	1.00
TSS total score	0.89		0.89		0.34		11.26	7.29	0–39	6.00	10.00	15.00
TSS fear subscale score		0.88		0.88	0.57		5.61	3.71	0–15	3.00	5.00	8.00
TSS coping subscale score		0.84		0.83	0.50		4.83	3.68	0–15	2.00	4.00	7.00
TSS Intrusive thoughts subscale score		0.46		0.48	0.23		0.82	1.30	0–9	0.00	0.00	1.00

*TSS* Tokophobia Severity Scale, *MIC* Mean inter–item correlation, *ITC* Corrected item-total correlation, *α* Cronbach’s alfa, *ω*  McDonald’s omega coefficient, *α IID* Cronbach’s alpha if item deleted, *ω IID* McDonald’s omega if item deleted, *P* Percentiles. For MIC and ITC, Spearman correlations were used, due to the ordinal nature of the variables

### Criterion (known-groups and concurrent) and convergent validity

#### Known-groups validity

##### *Nulliparous* non-pregnant women

Significant differences between groups were found for previous and current mental health problems, preferred childbirth, traumatic childbirth witness, been told a traumatic childbirth story. Women who do not plan to have children had higher TSS total scores than those who do not know if they want to have children (*p* < .001), and women who do not plan or do not know if they want to have children had higher scores than those who plan to have children (all *p* < .001). Women who perceive childbirth as a high-risk event reported higher TSS total scores than women who perceive childbirth as a medium-risk event (*p* < .001) and women who perceive childbirth as a high-risk or medium-risk event reported higher scores than women who perceive childbirth as a low-risk event (*p* < .001) (see Supplementary Table 7).

##### Pregnant women

Significant differences between groups were found for income, obstetric complications, exposure to potentially traumatic lifetime events, previous and current mental health problems, depressive or anxiety symptoms, and previous traumatic childbirth. Women reporting major fetal medical complications had higher TSS scores than those reporting minor (*p* = .011) or no fetal complications (*p* = .002) (see Supplementary Table 8).

Postpartum: Significant differences between groups were found for exposure to potentially traumatic lifetime events, depressive symptoms, and previous traumatic childbirth. Women who reported severe obstetric or fetal/neonatal medical complications, had higher TSS total scores than those who reported minor or no obstetric or fetal/neonatal medical complications (see Supplementary Table 9).

#### Concurrent validity

In nulliparous non-pregnant women, weak associations were found between TSS subscales/total score and depressive and anxiety symptoms. During pregnancy, moderate to strong positive correlations were found between TSS and depressive and anxiety symptoms. In the postpartum period, moderate to strong positive correlations were found between TSS subscales and depressive symptoms and moderate association with birth trauma perception, except for Lithuania with a weak association (see Supplementary Table 10).

### Convergent validity

Moderate to strong positive associations were found between TSS subscales/ total scores and fear of childbirth (FOBS score), in all countries but Lithuania in the postpartum, in which the association was weak (see Supplementary Table 10).

### Predictive validity

TSS Fear and Coping subscales during pregnancy predicted the type of birth - longitudinal data from Portugal. For each unit increase in the Fear subscale score, the likelihood of having a cesarean section increases by 16%, and for each unit increases in the Coping subscale score, the likelihood of having a cesarean section increases by 13% (see Table 6).

**Table 6:** Tokophobia Severity Scale predictive validity: total and subscales scores as predictors of type of birth, birth trauma perception and postpartum depressive and anxiety symptoms

Elective caesarian (vs. other)	χ^2^	df	$\:{R}_{N}^{2}$	B	Wald	OR	OR 95% CI
	1.19	1	0.01	-	-	-	-
TSS total score	-	-	-	0.03	1.21	1.03	0.98–1.08
Elective caesarian (vs. other)	8.02^*^	3	0.07	-	-	-	-
TSS Fear	-	-	-	0.15^*^	5.53	1.16	1.03–1.32
TSS Coping	-	-	-	-0.14^*^	3.93	0.87	0.76–0.99
TSS Intrusive	.	-	-	0.13	0.59	1.13	0.82–1.57
	*F*	*df1*,*df2*	*R²*	*B*	*95% CI*	*β*	*t*
Birth trauma perception	4.60^*^	1,171	0.03	-	-	-	-
TSS total score	-	-	-	0.06	0.01–0.11	0.16	2.14^*^
Birth trauma perception	3.19^*^	3,169	0.05	-	-	-	-
TSS Fear	-	-	-	0.03	-0.11-0.16	0.04	0.36
TSS Coping	-	-	-	0.19	0.04–0.33	0.24	2.49^*^
TSS Intrusive	-	-	-	-0.28	-0.66-0.10	− 0.13	-1.46
Postpartum depressive symptoms	32.34^***^	1,164	0.17	-	-	-	-
TSS total score	-	-	-	0.28	0.19–0.38	0.41	5.69^***^
Postpartum depressive symptoms	10.82^***^	3,162	0.17	-	-	-	-
TSS Fear	-	-	-	0.35	0.08–0.62	0.25	2.58^*^
TSS Coping	-	-	-	0.27	-0.01-0.54	0.19	1.94
TSS Intrusive	-	-	-	0.08	-0.62-0.79	0.02	0.23
Postpartum anxiety symptoms	32.28***	1,164	0.16	-	-	-	-
TSS total score	-	-	-	0.69	0.45–0.93	0.40	5.68***
Postpartum anxiety symptoms	10.81***	3,162	0.17	-	-	-	-
TSS Fear	-	-	-	0.78	0.14-0.1.43	0.24	2.40*
TSS Coping	-	-	-	0.49	− 0.17-1.15	0.14	1.46
TSS Intrusive	-	-	-	1.05	− 0.65-2.75	0.11	1.22

Portugal was the only country that evaluated TSS during pregnancy and used a subsample to evaluate measures of depression and anxiety during postpartum. Therefore, this Table only presents Portuguese data. Linear regressions were performed separately for each independent variable. Type of birth: 1- elective cesarean; *n* = 45), other : 0; *n* = 127);$\:{{\upchi\:}}_{HL}^{2}$: Hosmer-Lemeshow goodness of fit test;$\:{R}_{N}^{2}$=$\:Nagelkerke\:$R^2^ ; OR = Odds Ratio; TSS = Tokophobia Severity Scale; Postpartum anxiety symptoms were assessed using the State Anxiety Inventory (*n* = 173); Postpartum depression symptoms were assessed using the Edinburgh Postnatal Depression Scale (*n* = 166); Birth trauma perception (*n* = 166) was assessed and type of birth were collected from the sociodemographic questionnaire; *p < .05 **p < .01 ***p < .001

Both the TSS total score and the Coping subscale were predictors of higher birth trauma perception. Both TSS total score and the Fear subscale were predictors of higher depressive and anxiety symptom severity (see Table 6).

### Clinical validity

#### *Nulliparous* non-pregnant women

**Fig. 2:**
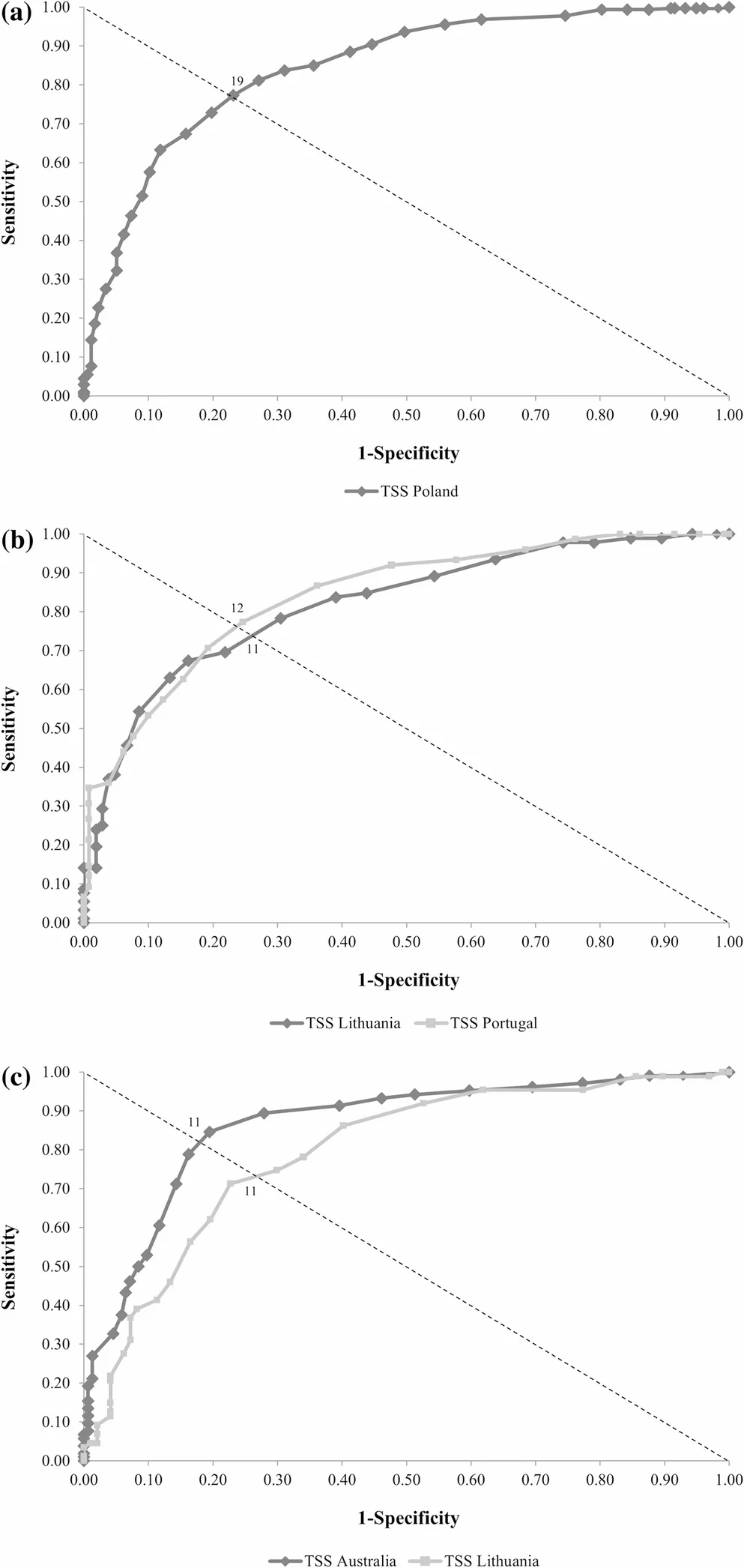
Receiver operating curve *to* determine the cut-off value of TSS with the best performance to identify women with and without clinically significant fear of childbirth. (**A**) Nulliparous non-pregnant women (**B**) Pregnant women (**C**) Women in postpartum. Notes: *TSS*  Tokophobia Severe Scale, *FOBS* Fear of Birth Scale, FOBS ≥ 50 = women with clinically significant fear of childbirth; (**A**) TSS Nulliparous non-pregnant women data, Poland: *n* = 313 women with FOBS ≥ 50 and *n* = 177 women with FOBS < 50; (**B**) TSS pregnancy data, Lithuania: *n* = 92 women with FOBS ≥ 50 and *n* = 105 women with FOBS < 50, Portugal: *n* = 75 women with FOBS ≥ 50 and *n* = 130 women with FOBS < 50; (**B**) TSS postpartum data, Australia: *n* = 104 women with FOBS ≥ 50 and *n* = 154 women with FOBS < 50, Lithuania: *n* = 87 women with FOBS ≥ 50 and *n* = 97 women with FOBS < 50

 Predictive ability to discriminate between women with (FOBS ≥ 50) versus without (FOBS < 50) clinically significant tokophobia symptoms was good (AUC = 0.84, 95% CI = 0.81 − 0.88). The optimal balance between sensitivity and specificity was at the TSS ≥ 19, at which 77.1% of women were correctly identified (see Table 7; Fig. 2).

**Table 7:** Tokophobia Severity Scale clinical validity: diagnostic performance for detection of clinical and significant fear of childbirth (FOBS) (*N* = 1585)

TSS	Poland (nulliparous non-pregnant, *n* = 490)				
Threshold	Sensitivity (95% CI)	Specificity (95% CI)	PPV (95% CI)	NPV (95% CI)	Accuracy (95% CI)
17	83.7 (80.4-87.0)	68.9 (64.8-73.0)	82.6 (79.2-86.0)	70.5 (66.4-74.5)	78.4 (74.7-82.0)
18	81.2 (77.7-84.6)	72.9 (68.9-76.8)	84.1 (80.9-87.3)	68.6 (64.4-72.7)	78.2 (74.5-81.9)
19	77.3 (73.6-81.0)	76.8 (73.1-80.6)	85.5 (82.4-88.6)	65.7 (61.5-69.9)	77.1 (73.4-80.8)
20	72.8 (68.9-76.8)	80.2 (76.7-83.8)	86.7 (83.7-89.7)	62.6 (58.3-66.9)	75.5 (71.7-79.3)
21	67.4 (63.7-71.6)	84.2 (80.9-87.4)	88.3 (85.5-91.1)	59.4 (55.0-63.7)	73.5 (70.0-77.4)
	Lithuania (pregnancy, *n* = 197)				
9	84.8 (80.4-89.2)	56.2 (50.1-62.3)	62.9 (57.0-68.8)	80.8 (76.0-85.6)	69.5 (63.9-75.1)
10	83.7 (79.2-88.2)	61.0 (55.1-67.0)	65.3 (59.5-71.1)	81.0 (76.2-85.8)	71.6 (66.1-77.1)
11	78.3 (73.3-75.7)	69.5 (63.9-75.1)	69.2 (63.6-74.8)	78.5 (73.5-83.5)	73.6 (68.2-79.0)
12	69.6 (64.0-75.2)	78.1 (73.1-83.1)	73.6 (68.2-79.0)	74.5 (69.2-89.8)	74.1 (68.8-79.4)
13	67.4 (61.7-73.1)	83.8 (79.3-88.3)	78.5 (73.5-83.5)	74.6 (69.3-79.9)	76.1 (70.9-81.3)
	Portugal (pregnancy, *n* = 205)				
10	92.0 (88.7-95.3)	52.3 (46.2-58.4)	52.7 (46.6-58.8)	91.9 (88.6-95.2)	66.8 (61.1-72.6)
11	86.7 (82.6-90.8)	63.8 (57.9-69.7)	58.0 (52.0-64.0)	89.2 (85.4-93.0)	72.2 (66.7-77.7)
12	77.3 (72.2-82.4)	75.4 (70.1-80.7)	64.4 (58.6-70.2)	85.2 (80.9-89.5)	76.1 (70.9-81.3)
13	70.7 (65.1-76.3)	80.8 (76.0-85.6)	67.9 (62.2-73.6)	82.7 (78.1-87.3)	77.1 (72.0-82.2)
14	62.7 (56.8-68.6)	84.6 (80.2-89.0)	70.1 (64.5-75.7)	79.7 (74.8-84.6)	76.6 (71.4-81.8)
	Australia (postpartum, *n* = 258)				
9	91.3 (87.9-94.8)	60.4 (54.4-66.4)	60.9 (54.9-66.9)	91.2 (87.7-94.7)	72.9 (67.5-78.3)
10	89.4 (85.7-93.2)	72.1 (66.6-77.6)	68.4 (62.7-74.1)	91.0 (87.5-94.5)	79.1 (74.1-84.1)
11	84.6 (80.2-89.0)	80.5 (75.7-85.4)	74.6 (69.3-80.0)	88.6 (84.7-92.5)	82.2 (77.5-86.9)
12	78.8 (73.9-83.8)	83.8 (79.3-88.3)	76.6 (71.4-81.8)	85.4 (81.1-89.7)	81.8 (77.1-86.5)
13	71.2 (65.6-76.7)	85.7 (81.4-90.0)	77.1 (72.0-82.2)	81.5 (76.8-86.2)	79.8 (74.9-84.7)
	Lithuania (postpartum, *n* = 184)				
9	78.2 (73.2-83.2)	66.0 (60.2-71.8)	67.3 (61.6-73.0)	77.1 (72.0-82.2)	71.7 (66.2-77.2)
10	74.7 (69.4-80.0)	70.1 (64.5-75.7)	69.1 (63.5-74.7)	75.6 (70.4-80.8)	72.3 (66.8-77.8)
11	71.3 (65.8-76.8)	77.3 (72.2-82.4)	73.8 (68.4-79.2)	75.0 (69.7-80.3)	74.5 (69.2-79.8)
12	62.1 (56.2-68.0)	80.4 (75.6-85.2)	74.0 (98.6-79.4)	70.3 (64.7-75.9)	71.7 (66.2-77.2)
13	56.3 (50.2-62.4)	83.5 (79.0-88.0)	75.4 (70.1-80.7)	68.1 (62.4-73.8)	70.7 (65.1-76.3)

*TSS* Tokophobia Severe Scale, *FOBS* Fear of Birth Scale, *PPV* Positive predictive value, *NPV* Negative predictive value, FOBS ≥ 50 = women in the clinical group; ^a^ = Polish nulliparous non-pregnant data, *n* = 313 women in the non-clinical group and *n* = 177 women in the clinical group; ^b^ = Lithuanian pregnancy data, *n* = 105 women in the non-clinical group and *n* = 92 women in the clinical group; ^c^ Portuguese pregnancy data, *n* = 130 women in the non-clinical group and *n* = 75 women in the clinical group ^d^ = Australian postpartum data, *n* = 154 women in the non-clinical group and *n* = 104 women in the clinical group; ^e^ = Lithuanian postpartum data, *n* = 97 women in the non-clinical group and *n* = 87 women in the clinical group

#### Pregnant women

Predictive ability to discriminate between women with (FOBS ≥ 50) versus those without (FOBS < 50) clinically significant tokophobia symptoms was good in women from Portugal (AUC = 0.84, 95% CI = 0.79 − 0.90) and from Lithuania (AUC = 0.82, 95% CI = 0.76 − 0.88). The optimal balance between sensitivity and specificity was at the TSS ≥ 11 for women from Lithuania and ≥ 12 for women from Portugal, at which 73.6% of women from Lithuania and 76.1% of women from Portugal were correctly identified (see Table 7; Fig. 2).

#### Postpartum

Predictive ability to discriminate between women with (FOBS ≥ 50) and without (FOBS < 50) clinically significant tokophobia symptoms was good in women from Australia (AUC = 0.86, 95% CI = 0.81 − 0.91) and acceptable in women from Lithuania (AUC = 0.79, 95% CI = 0.73 − 0.86). The optimal balance between sensitivity and specificity was at the TSS ≥ 11 for women from Australia and Lithuania. At this cut-off, 82.2% of women from Australia and 74.5% of women from Lithuania were correctly identified (see Table 7; Fig.2).

## Discussion

The main aim of this study is to analyze the psychometric properties of the TSS in nulliparous non-pregnant women, pregnant women, and women in the postpartum period using data from four countries: Australia, Lithuania, Poland, and Portugal. Our findings provided evidence on the psychometric properties of the TSS in childbearing-age women using data from four countries: Australia, Lithuania, Poland, and Portugal, suggesting that TSS items measure the same construct in the different childbearing-age periods and countries.

Our data supports a TSS three-factor model with 13 items and 3 subscales: fear, coping, and intrusive thoughts. This is similar to the three-factor model tested in a sample of Australian nulliparous, non-pregnant women by Martin et al. (2021), even though item 8 “*worry that I will not be in control of the medical procedures during my pregnancy and/or delivery*” saturates in the fear subscale whereas in our study saturates in the coping subscale. Additionally, Martin et al. (2021) found that the 3-factor model with 10 items (i.e. excluding items 1, 2, and 10 due to floor effects) offered a better fit. The fact that in our study we have included a sample of women from different countries - Australia, Lithuania, Poland and Portugal - and in different childbearing-age periods - nulliparous non-pregnant, pregnant and in the postpartum period -, may explain these differences. Furthermore, other sample characteristics that can impact mental health, such as sociodemographic or economic background, may differ between studies, which could also explain the differences.

The measurement invariance results suggested that the TSS has the same three dimensions and measures the same construct across different countries and childbearing-age periods, as we found no variation in the TSS factor structure according to the geographic location and the temporal proximity of the potentially phobic stimulus. This suggests its adequacy for different contexts, allowing comparisons between countries and childbearing-age periods.

The findings regarding the TSS total score reliability are consistent with the results of the original version (Wootton et al. 2020). Our results provide evidence of good to excellent internal consistency (George and Mallery 2019; Flora 2020) for nulliparous non-pregnant women, pregnant women, and women in the postpartum period, both for the total score, and Fear and Coping subscales, in all countries. Nevertheless, the TSS intrusive thoughts subscale had weak internal consistency (Cronbach’s alpha < 0.60), which is probably due to the fact that this subscale is composed of only three items with floor effects in our sample (items 7, 10, and 11), with a low proportion of participants indicating higher scores in this subscale, lacking variance, raising doubts about.

the validity of this subscale among nonclinical samples. Professionals and researchers using TSS should be aware of these subscale characteristics and use it with caution. Nevertheless, as removing these items did not improve the model fit in our sample, they were not removed. Further studies exploring the reliability of this subscale in clinical samples could be useful, since these symptoms may be more prevalent in more severe cases of fear of childbirth.

Evidence of known-group validity was found with sociodemographic, obstetric, and fetal/neonatal characteristics, without interaction effects with the country. Women’s perception of income was the only sociodemographic characteristic with criterion validity for TSS, exclusively in pregnancy. Women who reported income as below average or average (compared to above average) reported higher TSS scores, consistent with previous findings (Phunyammalee et al. 2019). Low socioeconomic status is linked to increased life stress and reduced access to healthcare, which can be associated with pregnancy complications without adequate prenatal care, potentially resulting in a perceived lack of control and fear regarding childbirth (Kim et al. 2018).

Women who experienced obstetric or fetal/neonatal complications (vs. those who did not) reported higher TSS scores in pregnancy and postpartum. These distressing experiences may exacerbate women’s apprehension about current and future births, contributing to a persistent cycle of fear and stress related to childbirth (Rúger-Navarrete et al. 2023). Nulliparous non-pregnant women, who expressed a preference for a future cesarean section (versus vaginal birth), heard traumatic childbirth stories, perceived childbirth as a high-risk event, or did not plan to have children, reported higher TSS scores. The preference for cesarean section is most likely indicative of fear conditioning related to hearing potentially traumatic childbirth stories, that can increase the perceived potential risk of childbirth (Wigert et al. 2020). These can lead to avoiding vaginal birth even if there is no medical indication, or hinder family planning (Kanellopoulos and Gourounti 2022; Striebich et al. 2018).

Our results also align with previous reports that link previous or current mental health problems (Dencker et al. 2019; Grundström et al. 2022), and previous traumatic birth experiences (Watson et al. 2021), with more tokophobia symptoms. These factors along with previous potentially traumatic events can influence an individual’s psychological response to childbirth by increasing vulnerability to fear and stress, as well as amplifying apprehensions about future births (Kranenburg et al. 2023).

TSS association with current birth trauma perception, and depressive and anxiety symptoms, are evidence of concurrent validity and are in line with findings from previous studies (Dencker et al. 2019; Gerges et al. 2024; Nath et al. 2021; Rondung et al. 2016). Perceiving childbirth as traumatic can impact mental health, as it often leads to heightened feelings of vulnerability and loss of control during labor, which may persist into the postpartum period (Rondung et al. 2016). Cognitive biases linked to depressive symptoms can result in negative emotions, reduced confidence in childbirth, increased insecurity, and heightened fear of subsequent childbirth (Nath et al. 2021). Also, strong fear associated with childbirth is associated with higher anxiety symptoms (Makara-Studzińska et al. 2022), consistent with the characterization of tokophobia within the scope of anxiety disorders (APA, 2022), in which anxiety is directly related to anticipatory fear and the perception of childbirth as threatening.

Evidence of TSS convergent validity was found in all childbearing age periods and countries, through the associations between TSS scores and fear of childbirth, assessed with the FOBS. Results of convergent validity suggested that the TSS measures a similar construct as FOBS. Fear of childbirth-related cognitions and behaviors assessed by TSS seem to be associated with fear of childbirth-related feelings assessed by FOBS. However, the correlation of the TSS intrusive thoughts subscale and FOBS showed a smaller effect size, which may be due to the items in this subscale being less aligned with the specific aspects of fear of childbirth measured by the FOBS (level of fear and worries).

Fear and Coping subscales in pregnancy predicted elective cesarean section, TSS total score and Coping subscale predicted birth trauma perception, and TSS total score and Fear subscale predicted depressive and anxiety symptoms in postpartum, which are evidence of predictive validity. This is in line with evidence that perceived lack of control during vaginal birth can intensify tokophobia symptoms and increase the odds of elective cesarean section (Hendrix et al. 2022; Sitras et al. 2017), and in women with more severe tokophobia symptoms, this fear may exacerbate their perception of birth trauma (Kitamura et al. 2024). Also, tokophobia can have a negative impact on women’s postpartum mental health when women view childbirth as a distressing experience because this distress can also disrupt the hormonal balance necessary for postpartum recovery, which may hinder physical healing and exacerbate mental health problems (Rouhe et al. 2011). TSS focus on worry - a transdiagnostic features of many mental health disorders (Akbari et al. 2018)-, can also be an advantage to predict anxiety and depression in the postpartum period.

Evidence of TSS’s clinical validity was found, with good classification accuracy - above 73.6% - to identify women with and without clinically significant tokophobia symptoms in nulliparous non-pregnant women, pregnant women, and women in the postpartum period, in Australia, Lithuania, Poland, and Portugal. This indicates good clinical validity. The optimal balance between sensitivity and specificity corresponds to different TSS cut-offs in different countries, which could be related to the demographic, obstetric and childbearing-age period differences between the countries. To our knowledge, this is the first study proposing country-specific cut-offs for the TSS to identify clinically significant tokophobia symptoms in different periods of childbearing age.

### Strengths and limitations

The main strength of this study is the use of multicountry data and heterogeneous samples, allowing the psychometric analysis of TSS in women from different childbearing-age periods across four countries. Although not available in all countries, the inclusion of sociodemographic, obstetric, and fetal/neonatal data, along with past and present mental health factors, and a measure of fear of childbirth is an added value to provide evidence of several psychometric properties of the TSS.

Different recruitment and data collection strategies, as well as differing inclusion criteria, contributed to heterogeneity in sample characteristics across countries (e.g. childbearing-age period), potentially impacting the study findings. Nonetheless, criterion validity analyses were conducted with consideration for these country-specific differences. A limitation of the Lithuanian subsample is that data were collected through online recruitment; however, the use of closed groups with membership verification and detailed cross-checking questions in the survey helped ensure data quality and minimize the risk of ineligible participants. Also, this study used the FOBS, which was not validated for nulliparous non-pregnant women. Another limitation is that the instructions provided to nulliparous non-pregnant women differed slightly from those given to pregnant and postpartum participants. This study would have benefited from additional information about why elective cesarean was chosen, for testing predictive validity, since understanding the motivations behind this decision could provide valuable insights into how tokophobia influences reproductive birth choices. Comparing the TSS with a gold standard measure of tokophobia in future studies would advance knowledge on clinical validity.

### Implications for clinical practice and research

The integration of tokophobia screening measures, such as the TSS, in healthcare settings could facilitate the early detection of women with clinically significant tokophobia symptoms, including those not pregnant, as well as the identification of at-risk women who could benefit from mental health interventions. The TSS demonstrated good psychometric properties, accurately identifying women with clinically significant tokophobia symptoms, and may be a valid and cost-effective screening tool for women at risk to be used as part of routine perinatal care appointments, in maternal and child healthcare settings. The use of TSS instead of a generalized anxiety disorder questionnaire would allow a timely and focused response to women’s care needs regarding either their reproductive choices or the approaching childbirth. This early screening could help mitigate some of the negative consequences of fear of childbirth, particularly in terms of mental health problems and future reproductive choices.

## Conclusion

TSS is a self-reported psychometrically strong measure that can effectively screen clinically significant tokophobia symptoms in childbearing women. Findings provided evidence of reliability, criterion, convergent, predictive, and clinical validity for the TSS in four countries either for nulliparous non-pregnant women, pregnant women, or women in the postpartum period. Overall, in some of the countries included in this study, this was the first time the psychometric characteristics of the TSS were examined. Future studies analyzing psychometric properties in other countries and populations (e.g. fathers, clinical samples, disadvantage samples) would advance knowledge.

## Supplementary Information

Below is the link to the electronic supplementary material.

Supplementary Material 1

## Data Availability

The data that supports the findings of this study are available on request from the corresponding author. The data are not publicly available due to privacy or ethical restrictions.
