# Supplementary material for: The tokophobia severity scale: a psychometric multicountry study with childbearing-age women
Source: Arch Womens Ment Health. 2026 Jun 18;29(4):95. doi: 10.1007/s00737-026-01736-9 (PMC13279276; doi:10.1007/s00737-026-01736-9)
Supplement: Supplementary file 1 — Supplementary Material 1 [file 737_2026_1736_MOESM1_ESM.docx]

| **Supplementary table 1.** Childbirth perception for *Polish nulliparous non-pregnant women (N = 559)* | | |
| --- | --- | --- |
|  | | Poland |
|  |  | Nulliparous non-pregnant women  (*n* = 559) |
| Plans to have children | Yes | 299 (53.5) |
|  | No | 109 (19.5) |
|  | Don’t know | 151 (27.0) |
|  | Missings | 0 (0.0) |
| Preferred type of childbirth | Vaginal | 335 (59.9) |
|  | Caesarean section | 224 (40.1) |
|  | Missings | 0 (0.0) |
| Perception of childbirth risk | Low risk* | 54 (9.7) |
|  | Medium risk** | 331 (59.2) |
|  | High risk*** | 174 (31.1) |
|  | Missings | 0 (0.0) |
| Witnessed a traumatic childbirth | Yes | 23 (4.1) |
|  | No | 536 (95.9) |
|  | Missings | 0 (0.0) |
| Been told a traumatic childbirth story | Yes | 304 (54.4) |
|  | No | 255 (45.6) |
|  | Missings | 0 (0.0) |
| Legend: *Low risk, The woman is able to give birth to the child and usually no medical interventions are needed to do so; **Medium risk, Childbirth is a normal physiological process, but medical interventions are necessary to manage the birth and provide the opportunity for the birth of a healthy baby; ***High risk, Childbirth is always high-risk for the baby and the mother | | |

| **Supplementary Table 2**. *Differences in sociodemographic, obstetric, fetal/neonatal, and mental health-related characteristics between countries by childbearing-age period data)* | | | | | | | |
| --- | --- | --- | --- | --- | --- | --- | --- |
|  | | Pregnant women | | | Postpartum women | | |
|  | | Lithuania  (*n* = 197)  *n* (%) | Portugal  (*n* = 353)  *n* (%) | p-value^a^ | Australia  (*n* = 292)  *n* (%) | Lithuania  (*n* = 184)  *n* (%) | p-value^a^ |
| Sociodemographic characteristics | | | | | | | |
| Ethnicity | Ethnic majority | - | 286 (87.2) |  | - | - |  |
|  | Ethnic minority | - | 9 (2.7) |  | - | - |  |
|  | Not sure | - | 33 (10.1) |  | - | - |  |
|  | Missings | - | 25 (7.1) |  | - | - |  |
| Perception of Income | Above average | 93 (47.2) | 68 (20.2) | Χ^2^(2) = 50.90^***^ | 51 (17.8) | 61 (33.2) | Χ^2^(2) = 30.50^***^ |
|  | Average | 101 (51.3) | 231 (68.8) |  | 195 (67.9) | 120 (65.2) |  |
|  | Below average | 3 (1.5) | 37 (11.0) |  | 41 (14.3) | 3 (1.6) |  |
|  | Missings | 0 (0.0) | 17 (4.8) |  | 5 (1.7) | 0 (0.0) |  |
| Marital status | Married/cohabiting | 189 (96.0) | 326 (94.2) | Χ^2^(1) = 0.76 | 260 (90.3) | 184 (100.0) | Χ^2^(1) = 19.02^***^ |
|  | Other* | 8 (4.0) | 20 (5.9) |  | 28 (9.7) | 0 (0.0) |  |
|  | Missings | 0 (0.0) | 7 (2.0) |  | 4 (1.4) | 0 (0.0) |  |
| Education | Higher education | 168 (85.3) | 219 (62.8) | Χ^2^(1) = 30.96^***^ | 212 (73.9) | 130 (70.7) | Χ^2^(1) = 0.58 |
|  | Other | 29 (14.7) | 130 (37.2) |  | 75 (26.1) | 54 (29.4) |  |
|  | Missings | 0 (0.0) | 4 (1.1) |  | 5 (1.7) | 0 (0.0) |  |
| *Obstetric and fetal/neonatal characteristics* | | | | | | | |
| Parity | Primiparous | 133 (67.5) | 210 (59.7) | Χ^2^(1) = 3.32 | 135 (46.9) | 109 (59.2) | Χ^2^(1) = 6.87^*^ |
|  | Multiparous | 64 (32.5) | 142 (40.3) |  | 153 (53.1) | 75 (40.8) |  |
|  | Missings | 0 (0.0) | 1 (0.3) |  | 4 (1.4) | 0 (0.0) |  |
| Previous pregnancy loss | Yes | 43 (21.8) | 97 (27.7) | Χ^2^(1) = 2.29 | 77 (50.3) | 51 (27.9) | Χ^2^(1) = 17.82^***^ |
|  | No | 154 (78.2) | 253 (72.3) |  | 76 (49.7) | 132 (72.1) |  |
|  | Missings | 0 (0.0) | 3 (0.8) |  | 139 (47.6) | 1 (0.5) |  |
| Obstetric complications | Yes, major complications | 4 (2.0) | 20 (5.7) | Χ^2^(2) = 7.61^*^ | 27 (9.2) | 15 (8.2) | Χ^2^(2) = 19.61^***^ |
|  | Yes, minor complications | 55 (27.9) | 120 (34.4) |  | 125 (42.8) | 44 (23.9) |  |
|  | No | 138 (70.1) | 209 (59.9) |  | 140 (47.9) | 125 (67.9) |  |
|  | Missings | 0 (0.0) | 4 (1.1) |  | 0 (0.0) | 0 (0.0) |  |
| Fetal/ neonatal medical complications | Yes, major complications | - | 11 (3.1) |  | 15 (5.2) | - |  |
|  | Yes, minor complications | - | 29 (8.3) |  | 70 (24.1) | - |  |
|  | No | - | 311 (88.6) |  | 206 (70.8) | - |  |
|  | Missings | - | 2 (0.6) |  | 1 (0.3) | - |  |
| Type of birth | Vaginal | - | - |  | 137 (46.9) | 138 (75.0) | Χ^2^(3) = 48.01^***^ |
|  | Assisted vaginal | - | - |  | 22 (7.5) | 8 (4.3) |  |
|  | Emergency Caesarian | - | - |  | 50 (17.1) | 28 (15.2) |  |
|  | Elective Caesarian Section | - | - |  | 83 (28.4) | 10 (5.4) |  |
|  | Missings | - | - |  | 0 (0.0) | 0 (0.0) |  |
| *Mental health-related factors* | | | | | | | |
| Potentially traumatic lifetime events | Yes | 90 (45.7) | 97 (29.2) | Χ^2^(1) = 14.67^***^ | 179 (61.3) | 97 (52.7) | Χ^2^(1) = 3.41 |
|  | No | 107 (54.3) | 235 (70.8) |  | 113 (38.7) | 87 (47.3) |  |
|  | Missings | 0 (0.0) | 21 (5.9) |  | 0 (0.0) | 0 (0.0) |  |
| Previous diagnosis of mental health problems | Yes | - | 77 (22.1) |  | 107 (36.6) | 11 (6.0) | Fisher = 65.30^***^ |
|  | No | - | 265 (75.9) |  | 182 (62.3) | 170 (92.4) |  |
|  | I don’t know | - | 7 (2.0) |  | 3 (1.0) | 3 (1.6) |  |
|  | Missings | - | 4 (1.1) |  | 0 (0.0) | 0 (0.0) |  |
| Current diagnosis of mental health problems | Yes | - | 39 (11.1) |  | - | 9 (4.9) |  |
|  | No | - | 294 (84.0) |  | - | 149 (81.0) |  |
|  | I don’t know | - | 17 (4.9) |  | - | 26 (14.1) |  |
|  | Missings | - | 3 (0.8) |  | - | 292 (61.3) |  |
| Clinically significant symptoms of depression^a^ | Yes | - | 41 (11.9) |  | 42 (14.5) | 37 (20.1) | Χ^2^(1) = 2.57 |
|  | No | - | 304 (88.1) |  | 248 (85.5) | 147 (79.9) |  |
|  | Missing | - | 8 (2.3) |  | 2 (0.7) | 0 (0.0) |  |
| Clinically significant symptoms of anxiety^b^ | Yes | - | 50 (27.0) |  | - | - |  |
|  | No | - | 135 (73.0) |  | - | - |  |
|  | Missing | - | 168 (47.6) |  | - | - |  |
| Previous traumatic childbirth | Yes | - | 35 (24.6) |  | 94 (61.8) | 23 (30.7) | Χ^2^(1) = 19.54^***^ |
|  | No | - | 107 (75.4) |  | 58 (38.2) | 52 (69.3) |  |
|  | Missings | - | 211 (59.8) |  | 140 (47.9) | 109 (59.2) |  |
| Age | *M (SD)* | 30.35 (4.52) | 33.05 (5.12) | *t*(541) = -6.16^***^; *d* = .55 | 32.42 (5.26) | 30.59 (4.50) | *t*(431) = 3.88^***^; *d* = .37 |
|  | *Missings* | 0 (0.0) | 7 (2.0) |  | 7 (2.4) | 0 (0.0) |  |
| Gestational weeks | *M (SD)* | 29.87 (5.89) | 33.44 (3.73) | *t*(326) = -7.30^***^; *d* = .73 | - | - |  |
|  | *Missings* | 0 (0.0) | 137 (38.8) |  | - | - |  |
| Gestational weeks at birth | *M (SD)* | - | 38.28 (1.92) |  | 38.52 (1.82) | 39.36 (2.13) | *t*(471) = -4.60^***^; *d* = .43 |
|  | *Missings* | - | 0 (0.0) |  | 3 (1.0) | 0 (0.0) |  |

| **Supplementary Table 3.** *Self-reported measures used in the study countries* | | | | | | |
| --- | --- | --- | --- | --- | --- | --- |
|  | Nulliparous non-pregnant women | Pregnant women | | Postpartum women | | |
|  | Poland | Lithuania | Portugal | Australia | Lithuania | Portugal |
| TSS | X | X | X | X | X | - |
| Sociodemographic, obstetric, fetal/neonatal, and mental health related data | X | X | X | X | X | X |
| EPDS | - | - | X | X | X | X |
| STAI-S | - | - | X | - | - | X |
| HADS | X | - | - | - | - | - |
| FOBS | X | X | X | X | X | - |
| Notes. TSS = Tokophobia Severity Scale; EPDS = Edinburgh Postnatal Depression Scale; STAI-S = State Anxiety Inventory; HADS = Hospital Anxiety and Depression Scale; FOBS = Fear of Birth Scale. | | | | | | |

| **Supplementary Table 4.**  *Differences in sociodemographic and clinical characteristics between pregnancy and postpartum* (in Portugal) | | | | |
| --- | --- | --- | --- | --- |
|  | | Portugal  (*n* = 166) | | Difference between pregnancy and postpartum data |
|  |  | Pregnancy | Postpartum | Statistics |
|  | | *n* (%) | *n* (%) |  |
| *Sociodemographic characteristics* | | | | |
| Ethnicity | Ethnic/racial majority | 140 (90.9) | 142 (92.2) | *MH* = 27.00 |
|  | Ethnic/racial minority | 3 (1.9) | 2 (1.3) |  |
|  | Not sure | 11 (7.1) | 10 (6.5) |  |
|  | Missings | 12 (7.2) | 12 (7.2) |  |
| Perception of Income | Above average | 34 (21.3) | 40 (25.0) | *MH* = 78.00 |
|  | Average | 113 (70.6) | 108 (67.5) |  |
|  | Below average | 13 (8.1) | 12 (7.5) |  |
|  | Missings | 6 (3.6) | 6 (3.6) |  |
| Marital status | Married/cohabiting | 159 (98.1) | 158 (97.5) | *MH* = 1.00 |
|  | Single/divorced/widow | 3 (1.9) | 4 (2.5) |  |
|  | Missings | 4 (2.4) | 4 (2.4) |  |
| Higher Education | Yes | 116 (70.7) | 115 (70.1) | *MH* = -1.00 |
|  | No | 48 (29.3) | 49 (29.9) |  |
|  | Missings | 2 (1.2) | 2 (1.2) |  |
| *Obstetric and fetal/neonatal characteristics* | | | | |
| Parity | Primiparous | 93 (56.4) | 93 (56.4) | *MH* = 0.00 |
|  | Multiparous | 72 (43.6) | 72 (43.6) |  |
|  | Missings | 1 (0.6) | 1 (0.6) |  |
| Previous pregnancy loss | Yes | 39 (23.6) | 45 (27.3) | *MH* = 6.00 |
|  | No | 126 (76.4) | 120 (72.7) |  |
|  | Missings | 1 (0.6) | 1 (0.6) |  |
| Obstetric complications | Yes, major complications | 11 (6.7) | 14 (8.5) | *MH* = 36.00 |
|  | Yes, minor complications | 54 (32.9) | 52 (31.7) |  |
|  | No | 99 (60.4) | 98 (59.8) |  |
|  | Missings | 2 (1.2) | 2 (1.2) |  |
| Fetal/ neonatal medical complications | Yes, major complications | 8 (4.8) | 11 (6.7) | *MH* = 11.00 |
|  | Yes, minor complications | 10 (6.1) | 17 (10.3) |  |
|  | No | 147 (89.1) | 137 (83.0) |  |
|  | Missings | 1 (0.6) | 1 (0.6) |  |
| Previous trauma | Yes | 51 (33.3) | 48 (31.4) | *MH* = -3.00 |
|  | No | 102 (66.7) | 105 (68.6) |  |
|  | Missings | 13 (7.8) | 13 (7.8) |  |
| Previous diagnosis of mental health problems | Yes | 36 (22.0) | 38 (23.2) | *MH* = 9.00 |
|  | No | 127 (77.4) | 124 (75.6) |  |
|  | I don’t know | 1 (0.6) | 2 (1.2) |  |
|  | Missings | 2 (1.2) | 2 (1.2) |  |
| Current diagnosis of mental health problems | Yes | 17 (10.4) | 14 (8.5) | *MH* = 13.00 |
|  | No | 141 (86.0) | 141 (86.0) |  |
|  | I don’t know | 6 (3.7) | 9 (5.5) |  |
|  | Missings | 2 (1.2) | 2 (1.2) |  |
| Clinical depression^a^ | Yes | 20 (12.1) | 17 (10.3) | *MH* = -3.00 |
|  | No | 145 (87.9) | 148 (89.7) |  |
|  | Missing | 1 (0.6) | 1 (0.6) |  |
| Clinical anxiety^b^ | Yes | 15 (26.8) | 7 (12.5) | *MH* = -8.00* |
|  | No | 41 (73.2) | 49 (87.5) |  |
|  | Missing | 110 (66.3) | 110 (66.3) |  |
| Previous traumatic childbirth | Yes | 18 (25.7) | 20 (28.6) | *MH* = 2.00 |
|  | No | 52 (74.3) | 50 (71.4) |  |
|  | Missings | 96 (57.8) | 96 (57.8) |  |
| Age | *n (%)* | 163 (98.2) | 163 (98.2) | *t*(162) = -6.10^***^ |
|  | *M (SD)* | 33.60 (4.86) | 33.85 (4.94) |  |

| **Supplementary Table 5.** Descriptive statistics of Edinburgh Postnatal Depression Scale total score, State Anxiety Inventory total score, and Fear of Birth Scale total scores | | | | | | | | | | |
| --- | --- | --- | --- | --- | --- | --- | --- | --- | --- | --- |
|  | EPDS | | | STAI-S | | | FOBS | | |  |
|  | *n* | *Range* | *M (SD)* | *n* | *Range* | *M (SD)* | *n* | *Range* | *M (SD)* |  |
| **Nulliparous non-pregnant women** |  |  |  |  |  |  |  |  |  |  |
| Poland | - | - | - | - | - | - | 490 | 0-100 | 57.11 (28.20) |  |
| **Pregnant women** |  |  |  |  |  |  |  |  |  |  |
| Lithuania | - | - | - | - | - | - | 197 | 0-100 | 47.60 (28.63) |  |
| Portugal | 345 | 0-25 | 7.21 (4.61) | 185 | 20-76 | 37.83 (11.22) | 205 | 0-100 | 38.15 (29.61) |  |
| **Postpartum women** | |  |  |  |  |  |  |  |  |  |
| Australia | 290 | 0-27 | 7.42 (5.54) | - | - | - | 258 | 0-100 | 40.91 (31.60) |  |
| Lithuania | 184 | 0-23 | 7.93 (5.31) | - | - | - | 184 | 0-100 | 46.10 (29.52) |  |
| Portugal subsample | 166 | 0-25 | 6.45 (4.99) | 163 | 20-79 | 34.52 (12.12) | - | - | - |  |
| *Notes*. EPDS= Edinburgh Postnatal Depression Scale; STAI-S= State Anxiety Inventory; TSS= Tokophobia Severe Scale. “-“: data not available | | | | | | | | | | |

| **Supplementary Table 6.** *Tokophobia Severity Scale factor structure: confirmatory factor analyses of the three-factor model proposed by Martin et al., 2022 (N = 1585)* | | | | | | | | | |
| --- | --- | --- | --- | --- | --- | --- | --- | --- | --- |
| Model | $\chi^{2}$ | $df$ | *p* | $\chi^{2}/df$ | RMSEA | 90% CI RMSEA | SRMR | CFI | TLI |
| M1 | 875.38 | 32 | < .001 | 27.36 | .13 | .12-.14 | .07 | .94 | .91 |
| M1b | 1248.59 | 19 | < .001 | 65.72 | .20 | .19-.21 | .08 | .91 | .87 |
| *Notes:* *M1: model with three factors and 10 items proposed by Martin et al (2022) M1b: M1 without the items with floor effects – items 7, 10 and 11* | | | | | | | | | |

| **Supplementary Table 7.** Known-groups validity of the Tokophobia Severity Scale in nulliparous non-pregnant women: differences according to sociodemographic, mental health-related factors (N = 559) | | | | | |
| --- | --- | --- | --- | --- | --- |
| Tokophobia Severity Scale scores | | | | | |
|  | | Poland (*n* = 559) | | Effects of the sociodemographic, obstetric and fetal/neonatal characteristics ^a^ | |
|  | | *n* (%) | *M (SD)* |  |  |
| Perception of Income | Above average | 95  (17.0) | 18.93  (9.23) | *F(2; 556)= 0.82;* $\eta_{p}^{2}$ *= .003* | |
|  | Average | 437 (78.2) | 20.13  (8.21) |  | |
|  | Below average | 27  (4.8) | 20.30  (8.55) |  | |
| Marital status | Married/cohabiting | 215 (38.5) | 20.27  (8.17) | *t(557)=-0.75; d = .065* | |
|  | Single/divorced/widow | 344 (61.5) | 19.72  (8.56) |  |  |
| Previous diagnosis of mental health problems | Yes | 166 (31.5) | 22.02  (7.83) | *t(525)=-4.11***; d = .385* | |
|  | No | 361 (68.5) | 18.80  (8.60) |  | |
| Current diagnosis of mental health problems | Yes | 208 (40.9) | 22.18  (7.85) | *t(507)=-5.17***; d = .466* | |
|  | No | 301 (59.1) | 18.34  (8.53) |  | |
| Plan to have children | Yes | 299 (53.5) | 17.89  (8.14) | *F(2; 556)= 26.11***;* $\eta_{p}^{2}$ *= .086* | |
|  | No | 109 (19.5) | 24.21  (8.36) |  | |
|  | Don’t know | 151 (27.0) | 20.89  (7.64) |  | |
| Prefer childbirth, if optional | Vaginal | 335 (59.9) | 17.32  (8.19) | *t(520)=-9.97***; d = .837* | |
|  | Caesarean section | 224 40.1) | 23.83  (7.13) |  | |
| Risk of childbirth perception | Low risk | 54  (9.7) | 13.11  (7.46) | *F(2; 556)= 62.08***;* $\eta_{p}^{2}$ *= .183* | |
|  | Medium risk | 331 (59.2) | 18.52  (7.96) |  | |
|  | High risk | 174 (31.1) | 24.74  (6.95) |  | |
| Traumatic childbirth witness | Yes | 23  (4.1) | 25.35  (7.42) | *t(557)=3.18**; d = .677* | |
|  | No | 536 (95.9) | 19.70  (8.38) |  | |
| Been told a traumatic childbirth story | Yes | 304 (54.4) | 21.12  (8.15) | *t(557)=3.67***; d = .312* | |
|  | No | 255 (45.6) | 18.52  (8.51) |  | |

| **Supplementary Table 8.** Known-groups validity of the Tokophobia Severity Scale in pregnant women: differences according to sociodemographic, obstetric, and fetal characteristics, and mental health-related factors, by country (N = 450) | | | | | | | | | | | |
| --- | --- | --- | --- | --- | --- | --- | --- | --- | --- | --- | --- |
|  | |  |  |  | | Tokophobia Severity Scale pregnancy scores | | | | | |
|  | | Lithuania  n=197 | | | Portugal  n=336 | | Total  n=533 | | Effects^a^ | | |
|  | | *n* (%) | *M (SD)* | | *n*  (%) | *M*  *(SD)* | *n* (%) | *M (SD)* | Sociodemographic, obstetric and fetal/neonatal characteristics | Country | Sociodemographic, obstetric, and fetal characteristics  *  Country |
| Perception of Income^b^ | Above average | *93*  *(47.2)* | *11.58 (6.63)* | | *68*  *(20.2)* | *11.06 (5.67)* | *161*  *(30.2)* | *11.36 (6.23)* | *F(1; 529)= 4.07*;* $\eta_{p}^{2}$ *= .008* | *F(1; 529)= 0.02;* $\eta_{p}^{2}$ *= .000* | *F(1; 529)= 0.82;* $\eta_{p}^{2}$ *= .002* |
|  | Below average or average | *104 (52.8)* | *12.33 (7.63)* | | *268 (79.8)* | *13.01 (6.79)* | *372*  *(69.8)* | *12.82 (7.03)* |  |  |  |
|  | Total by country | *197 (100.0)* | *11.97 (7.16)* | | *336 (100.0)* | *12.62 (6.61)* | *533 (100.0)* | *12.38 (6.82)* |  |  |  |
| Marital status | Married/cohabiting | *189 (95.9)* | *11.79 (6.85)* | | *326 (94.2)* | *12.46 (6.68)* | *515*  *(94.8)* | *12.21 (6.74)* | *H*(1) = 1.72 | *H*(1) = 0.23 | *H*(1) = 0.00 |
|  | Single/divorced/widow | *8*  *(4.1)* | *16.38 (12.42)* | | *20*  *(5.8)* | *13.80 (5.93)* | *28*  *(5.2)* | *14.54 (8.13)* |  |  |  |
|  | Total by country | *197 (100.0)* | *11.97 (7.16)* | | *346 (100.0)* | *12.54 (6.64)* | *543 (100.0)* | *12.33 (6.83)* |  |  |  |
| Higher Education | Yes | *168 (85.3)* | *11.95 (7.40)* | | *219 (62.8)* | *12.02 (6.45)* | *387*  *(70.9)* | *11.99 (6.87)* | *F(1; 542)= 1.18;* $\eta_{p}^{2}$ *= .002* | *F(1; 542)= 0.87;* $\eta_{p}^{2}$ *= .002* | *F(1; 542)= 0.71;* $\eta_{p}^{2}$ *= .001* |
|  | No | *29*  *(14.7)* | *12.14 (5.72)* | | *130 (37.2)* | *13.52 (6.85)* | *159*  *(29.1)* | *13.27 (6.66)* |  |  |  |
|  | Total by country | *197 (100.0)* | *11.97 (7.16)* | | *349 (100.0)* | *12.58 (6.63)* | *546 (100.0)* | *12.36 (6.83)* |  |  |  |
| Parity | Primiparous | 133 (67.5) | 12.49 (7.42) | | 210 (59.7) | 12.84 (6.58) | 343  (62.5) | 12.70 (6.91) | *F(1; 545)= 3.14;* $\eta_{p}^{2}$ *= .006* | *F(1; 545)= 1.59;* $\eta_{p}^{2}$ *= .003* | *F(1; 545)= 0.51;* $\eta_{p}^{2}$ *= .001* |
|  | Multiparous | 64 (32.5) | 10.91 (6.54) | | 142 (40.3) | 12.16 (6.69) | 206  (37.5) | 11.77 (6.66) |  |  |  |
|  | Total by country | 197 (100.0) | 11.97 (7.16) | | 352 (100.0) | 12.57 (6.62) | 549 (100.0) | 12.35 (6.82) |  |  |  |
| Previous pregnancy loss | Yes | 43 (21.8) | 12.07 (7.69) | | 97  (27.7) | 11.55 (6.08) | 140  (25.6) | 11.71 (6.59) | *F(1; 543)= 0.78;* $\eta_{p}^{2}$ *= .001* | *F(1; 543)= 0.10;* $\eta_{p}^{2}$ *= .000* | *F(1; 543)= 1.11;* $\eta_{p}^{2}$ *= .002* |
|  | No | 154 (78.2) | 11.95 (7.04) | | 253 (72.3) | 12.93 (6.79) | 407  (74.4) | 12.56 (6.89) |  |  |  |
|  | Total by country | 197 (100.0) | 11.97 (7.16) | | 350 (100.0) | 12.55 (6.62) | 547 (100.0) | 12.34 (6.82) |  |  |  |
| Obstetric complications^e^ | Yes | 59  (29.9) | 13.92 (8.03) | | 140 (40.1) | 13.83 (7.19) | 199  (36.4) | 13.85 (7.43) | *F(1; 542)= 14.49***;* $\eta_{p}^{2}$ *= .026* | *F(1; 542)= 0.15;* $\eta_{p}^{2}$ *= .000* | *F(1; 542)= 0.27;* $\eta_{p}^{2}$ *= .001* |
|  | No | 138  (70.1) | 11.14 (6.62) | | 209 (59.9) | 11.73 (6.06) | 347  (63.6) | 11.50 (6.29) |  |  |  |
|  | Total by country | 197  (100.0) | 11.97 (7.16) | | 349 (100.0) | 12.57 (6.61) | 546 (100.0) | 12.36 (6.81) |  |  |  |
| Fetal/ neonatal medical complications | Yes, major complications | - | - | | 11  (3.1) | 19.18 (5.88) | 11  (3.1) | 19.18 (5.88) | *F(2; 348)= 5.77**;* $\eta_{p}^{2}$ *= .032* |  |  |
|  | Yes, minor complications | - | - | | 29  (8.3) | 12.48 (7.20) | 29  (8.3) | 12.48 (7.20) |  |  |  |
|  | No | - | - | | 311 (88.6) | 12.36 (6.50) | 311  (88.6) | 12.36 (6.50) |  |  |  |
|  | Total by country | - | - | | 351 (100.0) | 12.59 (6.63) | 351 (100.0) | 12.59 (6.63) |  |  |  |
| Potentially traumatic events | Yes | *90*  *(45.7)* | *12.41 (6.88)* | | *97*  *(29.2)* | *14.10 (6.95)* | *187*  *(35.3)* | *13.29 (6.95)* | *F(1; 525)= 5.10*;* $\eta_{p}^{2}$ *= .010* | *F(1; 525)= 2.79;* $\eta_{p}^{2}$ *= .005* | *F(1; 525)= 0.99;* $\eta_{p}^{2}$ *= .002* |
|  | No | *107*  *(54.3)* | *11.61 (7.41)* | | *235 (70.8)* | *12.04 (6.38)* | *342*  *(64.7)* | *11.90 (6.71)* |  |  |  |
|  | Total by country | *197*  *(100.0)* | *11.97 (7.16)* | | *332 (100.0)* | *12.64 (6.61)* | *529 (100.0)* | *12.39 (6.82)* |  |  |  |
| Previous diagnosis of mental health problems | Yes | *-* | *-* | | *77*  *(22.5)* | *14.69 (7.35)* | *77*  *(22.5)* | *14.69 (7.35)* | *t(340)=-3.41***; d = .441* |  |  |
|  | No | *-* | *-* | | *265 (77.4)* | *11.82 (6.23)* | *265*  *(77.4)* | *11.82 (6.23)* |  |  |  |
|  | Total by country | *-* | *-* | | *342 (100.0)* | *12.46 (6.60)* | *342 (100.0)* | *12.46 (6.60)* |  |  |  |
| Current diagnosis of mental health problems | Yes | *-* | *-* | | *39*  *(11.7)* | *15.38 (7.15)* | *39*  *(11.7)* | *15.38 (7.15)* | *t(331)=-3.10**; d = .529* |  |  |
|  | No | *-* | *-* | | *294 (88.3)* | *11.96 (6.39)* | *294*  *(88.3)* | *11.96 (6.39)* |  |  |  |
|  | Total by country | *-* | *-* | | *333 (100.0)* | *12.36 (6.57)* | *333 (100.0)* | *12.36 (6.57)* |  |  |  |
| Clinically significant symptoms of depression^c^ | Yes | *-* | *-* | | *41*  *(11.9)* | *20.07 (6.90)* | *41*  *(11.9)* | *20.07 (6.90)* | *t(343) = -8.57***;d = 1.425* |  |  |
|  | No | *-* | *-* | | *304 (88.1)* | *11.54 (5.86)* | *304*  *(88.1)* | *11.54 (5.86)* |  |  |  |
|  | Total by country | *-* | *-* | | *345 (100.0)* | *12.55 (6.59)* | *345 (100.0)* | *12.55 (6.59)* |  |  |  |
| Clinically significant symptoms of anxiety^d^ | Yes | *-* | *-* | | *50*  *(27.0)* | *17.66 (6.30)* | *50*  *(27.0)* | *17.66 (6.30)* | *t(183) = -8.18**;d = 1.355* |  |  |
|  | No | *-* | *-* | | *135 (73.0)* | *10.40 (4.97)* | *135*  *(73.0)* | *10.40 (4.97)* |  |  |  |
|  | Total by country | *-* | *-* | | *185 (100.0)* | *12.36 (6.25)* | *185 (100.0)* | *12.36 (6.25)* |  |  |  |
| Previous traumatic childbirth | Yes | *-* | *-* | | *35*  *(24.6)* | *14.97 (6.63)* | *35*  *(24.6)* | *14.97 (6.63)* | *t(140) = -2.94**;d = .572* |  |  |
|  | No | *-* | *-* | | *107 (75.4)* | *11.24 (6.49)* | *107*  *(75.4)* | *11.24 (6.49)* |  |  |  |
|  | Total by country | *-* | *-* | | *142 (100.0)* | *12.16 (6.69)* | *142 (100.0)* | *12.16 (6.69)* |  |  |  |
| *Notes: ^a^* Non-parametric tests were performed when the assumptions of parametric tests were not met. In all other cases, parametric tests were reported as the parametric and non-parametric tests provided the same results; The non-parametric two-way ANOVA was *reported for marital status*, as the assumptions were not met and the non-parametric test results differed from the parametric results. ^b^ Below-average and the average groups were combined, as Lithuania had few women in the below-average group of perception of income. ^c^ Edinburgh Postnatal Depression Scale ≥ 13. ^d^ State Anxiety Inventory ≥ 45. ^e^ Categories of minor and major fetal/neonatal complications were combined, as Lithuania had few women in the major complications group; $\eta_{p}^{2}$ = partial eta squared “-“: data not available; * *p* < .050 ** *p* < .010 *** *p* < .001 | | | | | | | | | | | |

| **Supplementary Table 9.** Known-groups validity of the Tokophobia Severity Scale in women in postpartum: differences according to sociodemographic, obstetric, and fetal characteristics, and mental health-related factors, by country (N = 476) | | | | | | | | | | | |
| --- | --- | --- | --- | --- | --- | --- | --- | --- | --- | --- | --- |
|  | |  |  |  | | Tokophobia Severity Scale postpartum scores | | | | | |
|  | | Australia | | | Lithuania | | Total | | Effects^a^ | | |
|  | | *n (%)* | *M (SD)* | | *n (%)* | *M (SD)* | *n (%)* | *M (SD)* | Sociodemographic, obstetric, and fetal/neonatal characteristics | Country | Sociodemographic, obstetric, and fetal/neonatal characteristics  *  Country |
| Perception of Income^b^ | Above average | 51  (17.8) | 10.12 (6.67) | | 61 (33.2) | 12.26 (8.26) | 112 (23.8) | 11.29 (7.62) | *F*(1; 467)= 0.07; $\eta_{p}^{2}$ = .000 | *F*(1; 467)= 0.74; $\eta_{p}^{2}$ = .002 | *F*(1; 467)= 3.48; $\eta_{p}^{2}$ = .007 |
|  | Below average or Average | 236 (82.2) | 11.37 (7.39) | | 123 (66.8) | 10.59 (6.23) | 359 (76.2) | 11.10 (7.02) |  |  |  |
|  | Total by country | 287   (100.0) | 11.15 (7.27) | | 184 (100.0) | 11.14 (6.99) | 471 (100.0) | 11.15 (7.16) |  |  |  |
| Marital status^c^ | Married/cohabiting | 260 (90.3) | 11.12 (7.21) | | - | - | 260 (90.3) | 11.12 (7.21) | *t*(286)=-0.22; d = .043 |  |  |
|  | Single/divorced/widow | 28  (9.7) | 11.43 (7.85) | | - | - | 28  (9.7) | 11.43 (7.85) |  |  |  |
|  | Total by country | 288 (100.0) | 11.15 (7.26) | | - | - | 288 (100.0) | 11.15 (7.26) |  |  |  |
| Higher Education | Yes | 212 (73.9) | 11.00 (7.26) | | 130 (70.7) | 11.07 (6.83) | 342 (72.6) | 11.02 (7.09) | *F*(1; 467)= 0.19; $\eta_{p}^{2}$ = .000 | *F*(1; 467)= 0.00; $\eta_{p}^{2}$ = .000 | *F*(1; 467)= 0.01; $\eta_{p}^{2}$ = .000 |
|  | No | 75  (26.1) | 11.40 (7.21) | | 54 (29.3) | 11.31 (7.42) | 129 (27.4) | 11.36 (7.27) |  |  |  |
|  | Total by country | 287 (100.0) | 11.10 (7.23) | | 184 (100.0) | 11.14 (6.99) | 471 (100.0) | 11.12 (7.13) |  |  |  |
| Parity | Primiparous | 135 (46.9) | 10.47 (6.85) | | 109 (59.2) | 11.73 (7.34) | 244 (51.7) | 11.03 (7.09) | *F*(1; 468)= 0.02; $\eta_{p}^{2}$ = .000 | *F*(1; 468)= 0.02; $\eta_{p}^{2}$ = .000 | *F*(1; 468)= 4.02*; $\eta_{p}^{2}$ = .009 |
|  | Multiparous | 153 (53.1) | 11.75 (7.58) | | 75 (40.8) | 10.28 (6.39) | 228 (48.3) | 11.26 (7.23) |  |  |  |
|  | Total by country | 288 (100.0) | 11.15 (7.26) | | 184 (100.0) | 11.14 (6.99) | 472 (100.0) | 11.14 (7.15) |  |  |  |
| Previous pregnancy loss | Yes | 77  (50.3) | 11.38 (7.60) | | 51 (27.9) | 11.43 (6.94) | 128 (38.1) | 12.00 (7.32) | *F*(1; 332)= 0.06; $\eta_{p}^{2}$ = .000 | *F*(1; 332)= 0.34; $\eta_{p}^{2}$ = .001 | *F*(1; 332)= 0.42; $\eta_{p}^{2}$ = .001 |
|  | No | 76  (49.7) | 12.12 (7.59) | | 132 (72.1) | 11.08 (7.03) | 208 (61.9) | 11.46 (7.24) |  |  |  |
|  | Total by country | 153 (100.0) | 11.75 (7.58) | | 183 (100.0) | 11.18 (6.99) | 336 (100.0) | 11.44 (7.26) |  |  |  |
| Obstetric complications | Yes, major complications | 27  (9.2) | 17.48 (7.51) | | 15  (8.2) | 14.13 (9.23) | 42  (8.8) | 16.29 (8.21) | *F*(2; 470)= 9.38***; $\eta_{p}^{2}$ = .038 | *F*(1; 470)= 1.39; $\eta_{p}^{2}$ = .003 | *F*(2; 470)= 1.85; $\eta_{p}^{2}$ = .008 |
|  | Yes, minor complications | 125 (42.8) | 11.46 (7.66) | | 44 (23.9) | 10.64 (6.72) | 169 (35.5) | 11.25 (7.42) |  |  |  |
|  | No | 140 (47.9) | 10.03 (6.73) | | 125 (67.9) | 10.96 (6.75) | 265 (55.7) | 10.47 (6.74) |  |  |  |
|  | Total by country | 292 (100.0) | 11.33 (7.49) | | 184 (100.0) | 11.14 (6.99) | 476 (100.0) | 11.26 (7.29) |  |  |  |
| Fetal/ neonatal medical complications | Yes, major complications | 15  (5.2) | 18.80 (10.79) | | - | - | 15  (5.2) | 18.80 (10.79) | *F*(2; 288)= 8.22***; $\eta_{p}^{2}$ = .054 |  |  |
|  | Yes, minor complications | 70  (24.1) | 11.01 (7.27) | | - | - | 70  (24.1) | 11.01 (7.27) |  |  |  |
|  | No | 206 (70.8) | 10.95 (6.99) | | - | - | 206 (70.8) | 10.95 (6.99) |  |  |  |
| Type of birth | Vaginal | 137 (46.9) | 10.94 (6.89) | | 138 (75.0) | 10.91 (6.84) | 275 (57.8) | 10.92 (6.85) | *F*(3; 468)= 0.81; $\eta_{p}^{2}$ = .005 | *F*(1; 468)= 0.04; $\eta_{p}^{2}$ = .000 | *F*(3; 468)= 0.77; $\eta_{p}^{2}$ = .005 |
|  | Assisted vaginal | 22  (7.5) | 10.18 (7.40) | | 8  (4.3) | 13.50 (7.27) | 30  (6.3) | 11.07 (7.39) |  |  |  |
|  | Emergency Cesarean | 50  (17.1) | 12.20 (8.64) | | 28 (15.2) | 12.32 (7.83) | 78  (16.4) | 12.24 (8.31) |  |  |  |
|  | Elective Cesarean Section | 83  (28.4) | 11.76 (7.76) | | 10  (5.4) | 9.20 (6.36) | 93  (19.5) | 11.48 (7.63) |  |  |  |
|  | Total by country | 292 (100.0) | 11.33 (7.49) | | 184 (100.0) | 11.14 (6.99) | 476 (100.0) | 11.26 (7.29) |  |  |  |
| Potentially traumatic events | Yes | 179 (61.3) | 11.86 (7.59) | | 97 (52.7) | 12.56 (7.93) | 276 (58.0) | 12.11 (7.70) | *F*(1; 472)= 10.02***;* $\eta_{p}^{2}$ *=* .021 | *F*(1; 472)= 0.03*;* $\eta_{p}^{2}$ *=* .000 | *F*(1; 472)= 1.40*;* $\eta_{p}^{2}$ *=* .003 |
|  | No | 113 (38.7) | 10.50 (7.27) | | 87 (47.3) | 9.56 (5.37) | 200 (42.0) | 10.09 (6.52) |  |  |  |
|  | Total by country | 292 (100.0) | 11.33 (7.49) | | 184 (100.0) | 11.14 (6.99) | 476 (100.0) | 11.26 (7.29) |  |  |  |
| Previous diagnosis of mental health problems | Yes | 107 (37.0) | 13.22 (7.58) | | 11  (6.1) | 10.45 (5.66) | 118 (25.1) | 12.97 (7.45) | *F*(1; 466)= 0.82*;* $\eta_{p}^{2}$ *=* .002 | *F*(1; 466)= 0.56; $\eta_{p}^{2}$ *=* .001 | *F*(1; 466)= 2.39*;* $\eta_{p}^{2}$ = .005 |
|  | No | 182 (63.0) | 10.26 (7.20) | | 170 (93.9) | 11.22 (7.11) | 352 (74.9) | 10.73 (7.16) |  |  |  |
|  | Total by country | 289 (100.0) | 11.36 (7.47) | | 181 (100.0) | 11.18 (7.02) | 470 (100.0) | 11.29 (7.29) |  |  |  |
| *Clinically significant symptoms of depression^d^* | *Yes* | *42*  *(14.5)* | *17.31 (8.90)* | | *37 (20.1)* | *16.16 (7.99)* | *79*  *(16.7)* | *16.77 (8.45)* | *F*(1; 470)= 60.00^***^; $\eta_{p}^{2}$ = .113 | *F*(1; 470)= 0.90*;* $\eta_{p}^{2}$ = .002 | *F*(1; 470)= 0.16*;* $\eta_{p}^{2}$ = .000 |
|  | *No* | *248 (85.5)* | *10.35 (6.75)* | | *147 (79.9)* | *9.88 (6.12)* | *395 (83.3)* | *10.17 (6.52)* |  |  |  |
|  | *Total by country* | *290 (100.0)* | *11.36 (7.50)* | | *184 (100.0)* | *11.14 (6.99)* | *474 (100.0)* | *11.27 (7.30)* |  |  |  |
| *Previous traumatic childbirth* | *Yes* | *94*  *(61.8)* | *13.29 (7.01)* | | *23 (30.7)* | *12.26 (6.74)* | *117 (51.5)* | *13.09 (6.94)* | *F(*1; 223)= 11.50^***^; $\eta_{p}^{2}$ *=* .049 | *F*(1; 223)= 0.10*;* $\eta_{p}^{2}$ = .000 | *F*(1; 223)= 0.44; $\eta_{p}^{2}$ = .002 |
|  | *No* | *58*  *(38.2)* | *9.03 (7.69)* | | *52 (69.3)* | *9.40 (6.08)* | *110 (48.5)* | *9.21 (6.95)* |  |  |  |
|  | *Total by country* | *152 (100.0)* | *11.66 (7.54)* | | *75 (100.0)* | *10.28 (6.39)* | *227 (100.0)* | *11.21 (7.19)* |  |  |  |

Notes: ^a^ Non-parametric tests were performed when the assumptions of parametric tests were not met. Parametric tests were reported as the parametric and non-parametric tests provided the same results. ^b^ Below-average and the average groups were combined, as Lithuania had few women in the below-average group of perception of income; ^c^ Lithuania was removed from the analysis, as all women were married or cohabiting; ^d^ Edinburgh Postnatal Depression Scale ≥ 13.$\eta_{p}^{2}$ = partial eta squared; “-“: data not available; * *p* < .050 ** *p* < .010 *** *p* < .001

|  | | | | | | | | | | |
| --- | --- | --- | --- | --- | --- | --- | --- | --- | --- | --- |
| **Supplementary Table 10 -*.*** Concurrent and convergent validity of the Tokophobia Severity Scale: associations with depressive and anxiety symptoms, birth trauma perception, and fear of birth (N = 1585) | | | | | | | | | | |
|  |  | Nulliparous non-pregnant women | | | Pregnant women | | | Postpartum | | |
|  | TSS | Depressive Symptoms  N=559 | Anxiety Symptoms  N=559 | Fear of birth  N=490 | Depressive Symptoms  N=345 | Anxiety Symptoms  N=185 | Fear of birth^a^  N=402 | Depressive Symptoms^b^  N=474 | Birth trauma perception^c^  N=475 | Fear of birth^d^  N=442 |
| Poland | Total | .16^***^ | .16^***^ | .68^***^ | - | - | - | - | - | - |
|  | Fear | .12^***^ | .17^***^ | .57^***^ | - | - | - | - | - | - |
|  | Coping | .11^***^ | .13^**^ | .64^***^ | - | - | - | - | - | - |
|  | Intrusive | .22^***^ | .09^*^ | .46^***^ | - | - | - | - | - | - |
| Lithuania | Total | - | - | - | - | - | .68^***^ | - | - | - |
|  | Fear | - | - | - | - | - | .58^***^ | - | - | - |
|  | Coping | - | - | - | - | - | .63^***^ | - | - | - |
|  | Intrusive | - | - | - | - | - | .44^***^ | - | - | - |
| Portugal | Total | - | - | - | .56^***^ | .63^***^ | .68^***^ | - | - | - |
|  | Fear | - | - | - | .46^***^ | .51^***^ | .51^***^ | - | - | - |
|  | Coping | - | - | - | .51^***^ | .56^***^ | .68^***^ | - | - | - |
|  | Intrusive | - | - | - | .43^***^ | .38^***^ | .42^***^ | - | - | - |
| Total^1^ | Total | - | - | - | .56^***^ | .63^***^ | .67^***^ | - | - | - |
|  | Fear | - | - | - | .46^***^ | .51^***^ | .51^***^ | - | - | - |
|  | Coping | - | - | - | .51^***^ | .56^***^ | .65^***^ | - | - | - |
|  | Intrusive | - | - | - | .43^***^ | .38^***^ | .44^***^ | - | - | - |
| Australia | Total | - | - | - | - | - | - | .51^***^ | .46^***^ | .67^***^ |
|  | Fear | - | - | - | - | - | - | .46^***^ | .36^***^ | .56^***^ |
|  | Coping | - | - | - | - | - | - | .39^***^ | .46^***^ | .64^***^ |
|  | Intrusive | - | - | - | - | - | - | .41^***^ | .25^***^ | .36^***^ |
| Lithuania | Total | - | - | - | - | - | - | .51^***^ | .27^***^ | .50^***^ |
|  | Fear | - | - | - | - | - | - | .44^***^ | .19^***^ | .39^***^ |
|  | Coping | - | - | - | - | - | - | .46^***^ | .28^***^ | .52^***^ |
|  | Intrusive | - | - | - | - | - | - | .34^***^ | .16^***^ | .19^***^ |
| Total^2^ | Total | - | - | - | - | - | - | .51^***^ | .38^***^ | .60^***^ |
|  | Fear | - | - | - | - | - | - | .45^***^ | .29^***^ | .49^***^ |
|  | Coping | - | - | - | - | - | - | .41^***^ | .38^***^ | .59^***^ |
|  | Intrusive | - | - | - | - | - | - | .39^***^ | .22^***^ | .30^***^ |
| Note: ^a^  Lithuania n=197, Portugal n=205 ; ^b^ Australia n=290, Lithuania n=184; ^c^ Australia n=291, Lithuania n=184; ^d^ Australia n=258, Lithuania n=184; ^1^Lithuania and Portugal pregnant women; ^2^Australia and Lithuanian postpartum | | | | | | | | | | |
